# Proteasomes, Sir2, and Hxk2 Form an Interconnected Aging Network That Impinges on the AMPK/Snf1-Regulated Transcriptional Repressor Mig1

**DOI:** 10.1371/journal.pgen.1004968

**Published:** 2015-01-28

**Authors:** Yanhua Yao, Scott Tsuchiyama, Ciyu Yang, Anne Laure Bulteau, Chong He, Brett Robison, Mitsuhiro Tsuchiya, Delana Miller, Valeria Briones, Krisztina Tar, Anahi Potrero, Bertrand Friguet, Brian K. Kennedy, Marion Schmidt

**Affiliations:** 1 Department of Biochemistry, Albert Einstein College of Medicine, Bronx, New York, New York, United States of America; 2 Buck Institute, Novato, California, United States of America; 3 LCABIE UMR5254, Technopôle Hélioparc Pau Pyrénées, Pau, France; 4 Laboratoire de Biologie Cellulaire du Vieillissement, UR4-IFR83, Université Pierre et Marie Curie-Paris 6, Paris, France; Stanford University Medical Center, UNITED STATES

## Abstract

Elevated proteasome activity extends lifespan in model organisms such as yeast, worms and flies. This pro-longevity effect might be mediated by improved protein homeostasis, as this protease is an integral module of the protein homeostasis network. Proteasomes also regulate cellular processes through temporal and spatial degradation of signaling pathway components. Here we demonstrate that the regulatory function of the proteasome plays an essential role in aging cells and that the beneficial impact of elevated proteasome capacity on lifespan partially originates from deregulation of the AMPK signaling pathway. Proteasome-mediated lifespan extension activity was carbon-source dependent and cells with enhancement proteasome function exhibited increased respiratory activity and oxidative stress response. These findings suggested that the pro-aging impact of proteasome upregulation might be related to changes in the metabolic state through a premature induction of respiration. Deletion of yeast AMPK, *SNF1*, or its activator *SNF4* abrogated proteasome-mediated lifespan extension, supporting this hypothesis as the AMPK pathway regulates metabolism. We found that the premature induction of respiration in cells with increased proteasome activity originates from enhanced turnover of Mig1, an AMPK/Snf1 regulated transcriptional repressor that prevents the induction of genes required for respiration. Increasing proteasome activity also resulted in partial relocation of Mig1 from the nucleus to the mitochondria. Collectively, the results argue for a model in which elevated proteasome activity leads to the uncoupling of Snf1-mediated Mig1 regulation, resulting in a premature activation of respiration and thus the induction of a mitohormetic response, beneficial to lifespan. In addition, we observed incorrect Mig1 localization in two other long-lived yeast aging models: cells that overexpress *SIR2* or deleted for the Mig1-regulator *HXK2*. Finally, compromised proteasome function blocks lifespan extension in both strains. Thus, our findings suggest that proteasomes, Sir2, Snf1 and Hxk2 form an interconnected aging network that controls metabolism through coordinated regulation of Mig1.

## Author Summary

Advanced cellular age is associated with decreased efficiency of the proteostasis network. The proteasome, a protease in the cytoplasm and nuclei of eukaryotic cells, is an important component of this network. Recent studies demonstrate that increased proteasome capacity has a positive impact on longevity. The underlying mechanisms, however, have not been fully identified. Here we report that proteasomes are involved in regulating the AMP-activated kinase (AMPK) pathway and thus participate in correct metabolic adaptation. We find that Mig1, a transcriptional repressor downstream of yeast AMPK, Snf1, is a proteasome target and a negative regulator of lifespan. Increased proteasome activity results in enhanced turnover and incorrect localization of Mig1. The reduced Mig1 levels result in the induction of respiration and upregulation of the oxidative stress response. Premature Mig1 inactivation is also observed in two additional long-lived strains that overexpress *SIR2* or are deleted for *HXK2* and lifespan extension in both strains requires correct proteasome function. Our results uncover an interconnected network comprised of the proteasome, Sir2 and AMPK/Hxk2 signaling that impacts longevity through regulation of Mig1 and modulates respiratory metabolism. Mechanistic information on the cross-communication between these pathways is expected to facilitate the identification of novel pro-aging interventions.

## Introduction

Many genetic and pharmacological interventions that affect lifespan in model organisms modulate the respiratory capacity of the mitochondria and change the metabolic status of cells. The conserved pro-longevity effects of caloric restriction (CR) have been related to optimal mitochondrial function [1, 2, 3]. Paradoxically, however, mitochondria are the main source for reactive oxygen species (ROS) that covalently modify and inactivate cellular macromolecules. ROS-induced damage to proteins, lipids and nucleotides has been proposed to drive the age-induced decline in cellular function and is closely linked to a range of age-related chronic disease states. Yet strategies to reduce ROS have led to inconclusive effects on lifespan in different model organisms. On the other hand, several recent reports indicate that a moderate increase in ROS production in response to increased respiration activates a stress response that has a beneficial impact on lifespan [1, 4, 5, 6, 7]. This has given rise to the mitohormesis hypothesis, which posits that a low dose oxidative stress induced by increased respiratory activity has a beneficial impact on longevity.

Pathways that regulate respiration, the metabolic state and the cellular stress response play a pivotal role in the aging process. The target of rapamycin (TOR) pathway is involved in regulating the cellular response to nutrients, and has a well-studied role in the regulation of lifespan. In fact, apart from CR, treating cells with the TORC1 inhibitor rapamycin is the most successful pharmacological intervention to date that promotes longevity [8, 9, 10, 11]. TORC1 is active under optimal growth conditions and promotes ribosome biogenesis, protein translation, and cell growth. TORC1 activity is antagonized by the AMPK/Snf1 signaling pathway. While TORC1 signaling is inhibited upon nutrient limitation and stress, AMPK/Snf1s are activated by these conditions [12] AMPK also plays an active role in the termination of TORC1 signaling. Many of the cellular consequences of TOR inactivation are directly regulated by the activity of AMPKs, such as phosphorylation of transcriptional regulators involved in optimizing mitochondrial function and stress tolerance [13, 14] or the induction of autophagy via ULK1 phosphorylation [15]. Since AMPKs serve as fuel gauges and are highly conserved in eukaryotic cells [16] they are primed to play an important role in longevity and the cellular response to CR [17]. The impact of AMPK on longevity in mammals, however, is largely unexplored [17]. Several previous studies in different model organisms support the hypothesis that AMPK/Snf1 activation positively impacts lifespan [18]. In *C. elegans*, overexpression of the worm ortholog AAK-2 promotes lifespan extension [19] and AMPK activity is increased under CR conditions [1]. Tissue-specific knock-down of the *Drosophila* ortholog decreased lifespan [20]. However, findings in yeast remain controversial with Snf1 either promoting or decreasing lifespan [2, 21, 22]. Clearly, additional studies are required to elucidate the precise role of AMPKs in aging cells.

The cellular response induced by increased respiration and oxidative stress includes repair systems such as antioxidant enzymes, DNA-repair pathways, chaperones and the proteolytic systems. Importantly, genetic upregulation of many of these systems can extend lifespan in different organisms. As essential arms of the cellular stress response, the proteolytic systems prevent the accumulation of aggregation prone proteins and recycle amino acids for new synthesis. The proteasome is the primary protease in the cytoplasm and nuclei of eukaryotic cells [23]. Several recent studies demonstrated that improved proteolytic capacity through genetic upregulation of the proteasome increases lifespan. Using *Saccharomyces cerevisiae* as a model system we characterized the replicative lifespan (RLS) of cells with increased or decreased proteasome pools and found a positive correlation between proteasome activity and replicative lifespan in yeast [24]. Beneficial effects on lifespan and stress response upon upregulation of individual proteasome subunits were also observed in *C. elegans* and *Drosophila* [25, 26]. Furthermore, all exceptionally long-lived organisms examined exhibit increased proteasome activity [27, 28, 29].

The underlying mechanisms for proteasome-mediated lifespan extension remain to be elucidated. We found that the increased lifespan in cells with elevated proteasome activity correlated with improved tolerance towards proteotoxic stress and reduced aggregation of a neurotoxic protein expressed in yeast [24]. Additionally, a recent study reported reduced aggregation of endogenous proteins in aged mother cells upon upregulation of proteasome activity [30]. Thus, the beneficial impact of increased proteasomal activity on longevity is at least partially related to improved protein homeostasis. Yet, proteasomes influence cellular functions at many levels. In addition to being an essential component of the proteostasis, network they also regulate a large number of pathways through the timed degradation of proteins relevant to signal transduction and gene transcription in eukaryotic cells [31].

Here we report that the proteasome is involved in regulating AMPK/Snf1 signaling and that this activity impacts longevity in *S. cerevisiae*. Components of the Snf1 pathway are required for the pro-longevity effect in cells with elevated proteasome capacity. These cells exhibit decreased levels of Mig1, the major Snf1-regulated transcriptional repressor that prevents the expression of genes required for the induction of respiration under optimal growth conditions. In addition, Mig1 is relocalized to the mitochondria. Consistent with reduced Mig1 activity, signature genes, normally repressed by Mig1 under favorite growth conditions, are induced in cells with elevated proteasome activity resulting in increased respiratory capacity. We propose that the premature increase in respiration induces a hormetic response that contributes to lifespan extension in cells with increased proteasome activity. However, complete loss of Mig1 in cells with increased proteasome activity abrogates proteasome-mediated lifespan extension, suggesting that in addition to repression of genes required for respiration, Mig1 must have an additional function during respiratory growth that positively contributes to longevity. This hypothesis is supported by the observation that a *MIG1* mutant that is unable to exit the nucleus exhibits a short lifespan and prevents proteasome-mediated lifespan extension. Since we observed co-localization of Mig1 with mitochondria and increased mitochondrial fragmentation in the absence of Mig1, we propose that cytoplasmic Mig1 positively impacts respiration. *MIG1* deletion also abrogated lifespan extension conferred by overexpression of *SIR2*, emphasizing that AMPK/Snf1 signaling impinges on other longevity pathways. Lastly, reduced proteasome activity prevented lifespan extension in *SIR2* overexpressing strains and in a genetic model for caloric restriction through deletion of *HXK2*. Collectively, the results obtained provide evidence for an interconnected aging network formed by the proteasome, AMPK/Hxk2 signaling and Sir2 that impinges on Mig1 to regulate respiration and thus longevity.

## Results

### Proteasome-mediated lifespan extension is attenuated under growth conditions that restrict cells to either fermentative or respiratory metabolism

**Figure 1:**
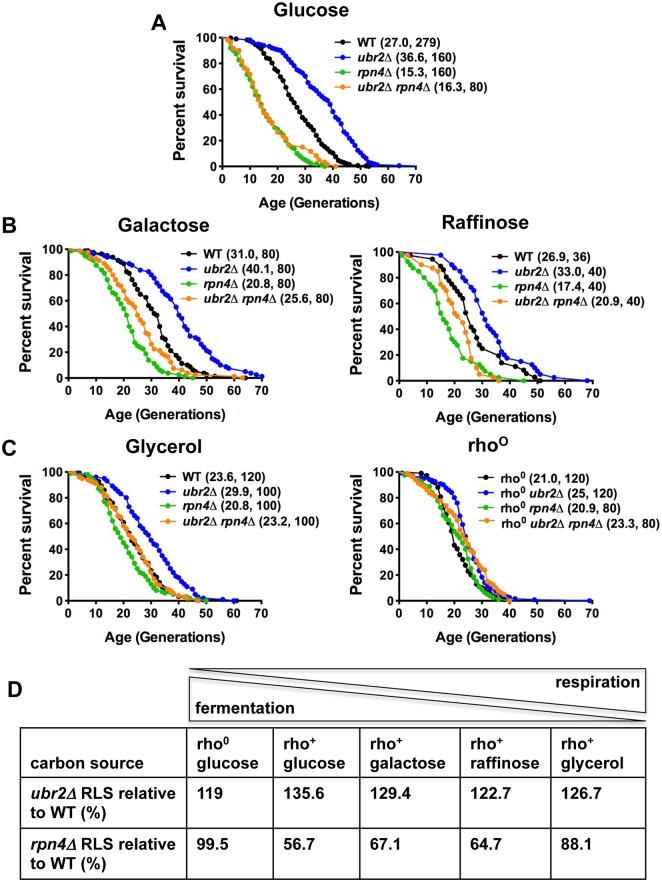
Proteasome-mediated impact on RLS is attenuated in response to the carbon source. (A) Longevity correlates with proteasome abundance. Survival curves of cells with increased (*ubr2Δ*) or reduced proteasome capacity (*rpn4Δ* or *rpn4Δ ubr2Δ*) grown in glucose media at 30°C. (B) Proteasome-mediated lifespan extension varies with the carbon-source. Survival curves of the strains used in (A) grown on media with galactose or raffinose as carbon source. (C) The impact of varying proteasome levels are largely abrogated on the non-fermentable carbon source glycerol (left panel) or in cells devoid of functional mitochondria (rho^0^, right panel). Mean lifespan and cell counts are shown in parenthesis. A statistical analysis of the data is summarized in S3 Table. (D) Relative lifespan extension or shortening in proteasome mutants in response to different carbon sources. The ratio between respiration and fermentation induced by the different carbon sources is indicated on top.

Deletion of the proteasome-related transcription factor *RPN4* results in cells with a low, non-adaptive proteasome pool [32], while loss of the ubiquitin ligase Ubr2 has the opposite effect because Ubr2 marks Rpn4 for proteasomal degradation [24]. Cells with increased proteasome activity (*ubr2Δ)* exhibit increased replicative lifespan (RLS), while a reduction in proteasome levels (*rpn4Δ or rpn4Δ ubr2Δ)* reduces RLS (Fig. 1A) [24]. To explore proteasome-mediated effects on longevity under different environmental conditions, we compared the lifespan of proteasome mutants on carbon sources that induce different ratios of fermentation versus respiration. While glucose leads to ATP production largely through fermentation, glycerol fully induces respiratory metabolism. A more balanced ratio of fermentation and respiration is observed in cells grown on galactose and raffinose [14]. Interestingly, carbon sources that induce respiratory metabolism blunted the RLS differences observed in proteasome mutants. Mean lifespan extension in *ubr2Δ* cells relative to wild type on glucose was 37%, on galactose 29%, on raffinose 23% and on glycerol 26% (Fig. 1A–D). Thus, lifespan extension was lower on carbon sources that prevent fermentation. Similarly, the lifespan shortening observed in *rpn4Δ* cells was largely attenuated on media that enforce respiration (Fig. 1A–D).

Based on these findings, we speculated lifespan extension by *ubr2Δ* would be maximized in rho^0^ cells, which lack the mitochondrial genome and the ability to respire as a result, since these cells are forced to ferment. In contrast, lifespan extension was reduced in this background (only 19% mean RLS extension). (Fig. 1C, right panel). Interestingly, *rpn4Δ* cells were not short-lived in this context, indicating that impaired proteasome function is not an impediment to longevity in cells unable to respire. These results suggest that increased proteasome activity is advantageous under growth conditions that allow both fermentation and respiration, where the cell may dynamically shift between modes of metabolism. These observations point to a link between proteasome-mediated lifespan extension and respiratory metabolism.

### Altered proteasome abundance impacts mitochondrial morphology and respiratory capacity

**Figure 2:**
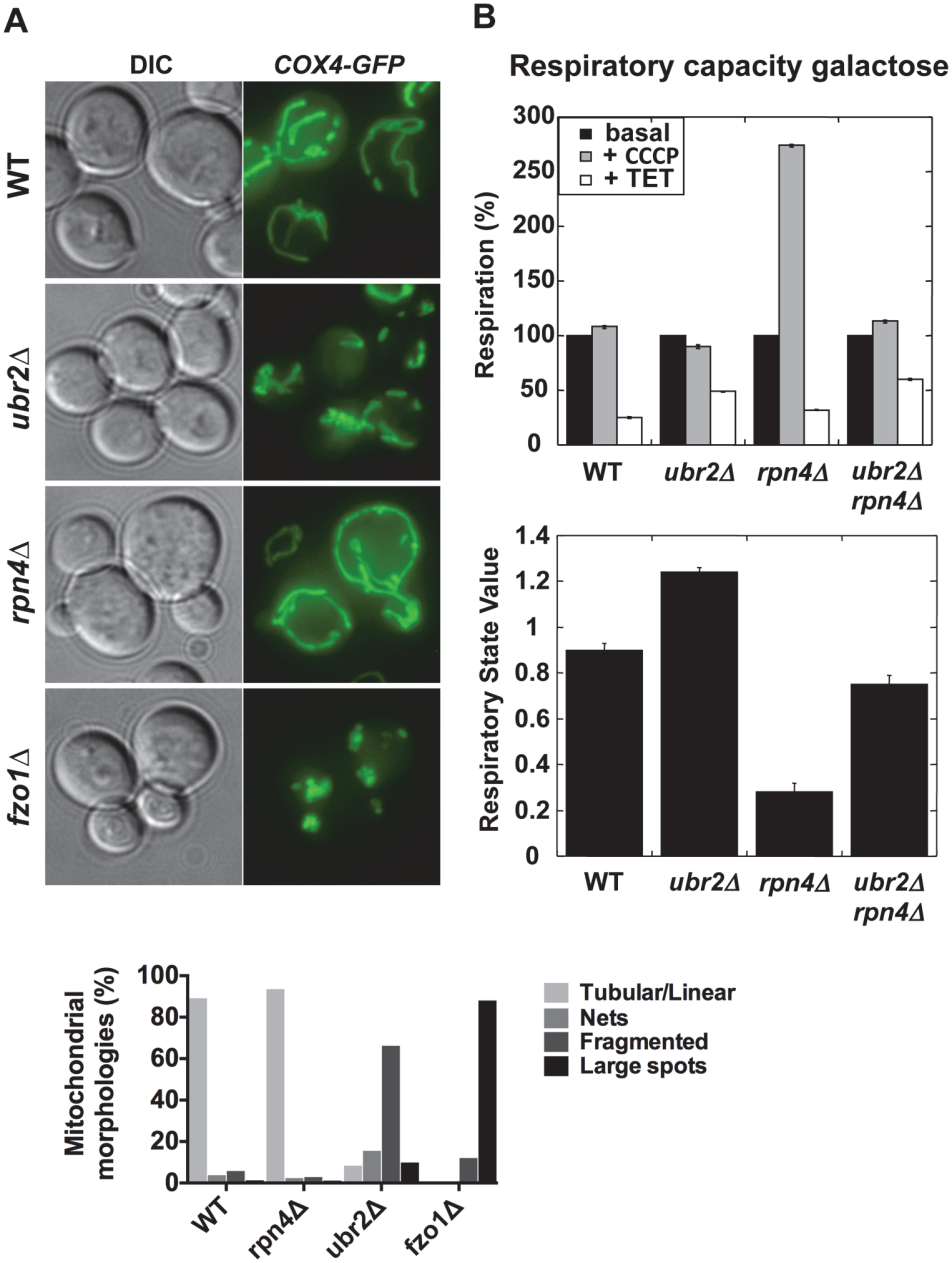
Altered mitochondrial morphology and activity in cells with varying proteasome capacity. (A) Increased proteasome abundance results in hyper-fragmented mitochondria, while reduced proteasome abundance causes hyper-fusion. To visualize mitochondria GFP was tagged with a mitochondrial import signal and introduced in cells with increased (*ubr2Δ*), decreased (*rpn4Δ*) proteasome capacity and in cells deleted for *FZO1*. Projected sequential Z-stacks fluorescence images are presented. DIC: differential interference contrast. ~ 200 cells were analyzed for each strain by visual inspection and their distinct mitochondrial morphologies is presented in the lower panel. (B) Mitochondrial activity correlates with proteasome abundance when cells are grown in galactose. The respiratory capacity of isolated mitochondria from cells with increased (*ubr2Δ*) or reduced proteasome capacity (*rpn4Δ* or *rpn4Δ ubr2Δ*) grown in galactose was tested using an Oroboros high-resolution respirometer. Upper Panel: Basal respiration was measured in the absence of drugs. To determine maximal respiration the uncoupling reagent carbonyl cyanide *m*-chlorophenyl hydrazone (CCCP) was added, which dissipates the proton gradient across the mitochondrial membrane. Leak flux was analyzed in the presence of triethyltin bromide (TET), an inhibitor of ATP-synthase. Lower panel: RSV is defined as (JO_basal_–JO_TET_)/ (JO_CCCP_–JO_TET_) and represents the percentage of stimulation of oxidative phosphorylation compared to the basal respiration capacity.

Proteasomes provide a quality control function at the outer membrane of the mitochondria by degrading damaged mitochondrial proteins [33]. Furthermore, proteasomal degradation is required for the regulation of mitochondrial morphological changes. Proteasomal degradation of mitofusins/Fzo1 prevents a hyperfused network and excessive fission under oxidative stress is prevented via Drp1/Dnm1 degradation [34, 35, 36, 37]. Given the variation of proteasome mutant RLSs under different metabolic conditions we investigated the morphology and activity of mitochondria. We visualized the mitochondrial network in cells with elevated or decreased proteasome capacity. We observed increased fragmentation in *ubr2Δ* cells and increased fusion in *rpn4Δ* cells both in glucose (Fig. 2A) and galactose (S1 Fig.). The morphological differences in the mitochondrial network in cells with increased or decreased proteasome activity correlate with Fzo1 levels, consistent with previous reports [34, 35, 38]. Decreased Fzo1 abundance was observed in cells with elevated proteasome activity, while reduced proteasome abundance resulted in increased Fzo1 levels (S2A Fig.).

Deletion of *FZO1* results in loss of mitochondrial DNA and negatively impacts the respiratory capacity of cells [39]. To test the respiratory state of *ubr2Δ* or *rpn4Δ* and *rpn4Δ ubr2Δ* cells, we analyzed O_2_ consumption of cells grown in galactose or lactate in an Oroboros respirometer, which records mitochondrial oxygen consumption [40]. Growth on galactose induces an intermediary metabolic state with roughly equal contributions from glycolysis and respiration, while cells grown on lactose exclusively respire [14]. Respiration was measured at basal levels as well as in the presence of *m*-chlorophenyl hydrazone (CCCP) and triethyltin bromide (TET). Addition of CCCP dissipates the proton gradient across the mitochondrial membrane, which has a crucial function in regulating the capacity of respiratory chain complexes. Removing the membrane potential by addition of CCCP allows for the recording of maximum respiration. Respiration was also measured in the presence of the ATP-synthase inhibitor triethyltin bromide (TET), which assesses non-phosphorylating respiration caused by proton leakage across the inner mitochondrial membrane (LEAK flux). From these data, we calculated an *in vivo* respiratory state value (RSV; [41]) in cells grown in galactose. Contrary to our expectation based on the fragmented mitochondrial phenotype, we found that cells lacking *UBR2* exhibit a ~ 30% increase in respiratory chain activity, while deletion of *RPN4* led to a reduction in respiration (Fig. 2B). In the presence of lactate, however, proteasome mutants only marginally affected respiratory capacity (S2B Fig.), presumably because cells grown on lactate exhibit already maximum induction of respiratory metabolism. These findings indicate that loss of *UBR2* does not affect the maximal respiratory capacity but instead shifts cells toward respiratory metabolism in environments where cells normally rely on both metabolic sources of ATP. In contrast, the non-adaptable proteasome pool in the *rpn4∆* mutant skews metabolism away from respiration.

**Figure 3:**
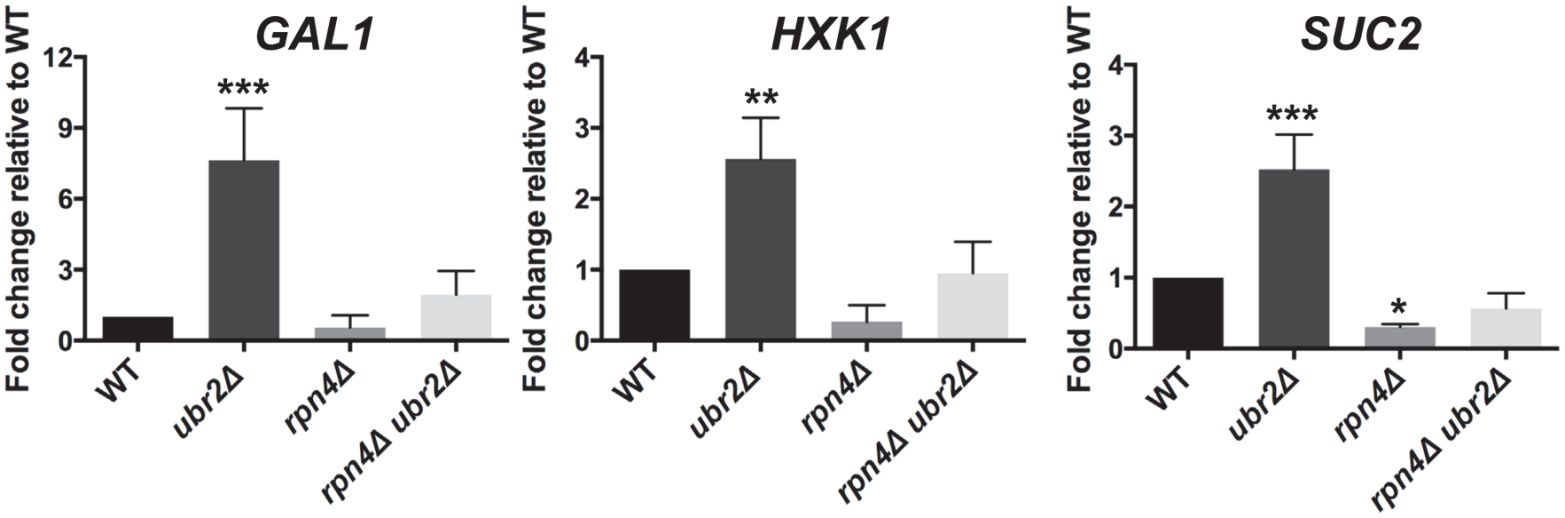
Cells with increased proteasome abundance exhibit features indicative of prematurely activated Snf1/AMP kinase signaling. The mRNA levels of the glucose repressed genes *GAL1*, *HXK1* and *SUC2* were tested via quantitative RT-PCR. mRNA was prepared after cells were grown in galactose for 4 h in WT, *rpn4Δ*, *ubr2Δ, or rpn4Δ ubr2Δ* cells. The data were corrected for the housekeeping gene *ACT1* and presented relative to WT expression as the mean +/- SEM of three biological replicates. *P*-values represent the statistical significance relative to WT expression and were assessed by an Ordinary one-way Annova using the GraphPad Prism software. *P*-values: * p < 0.05, ** p < 0.01, *** p < 0.001.

A typical response in yeast after cells switch from fermentation to respiration is the rapid nuclear accumulation of the stress and nutrient responsive transcription factor Msn2 [42], since this metabolic switch results in oxidative stress due to increased respiratory chain activity. We therefore reasoned that the increased respiratory activity of *ubr2Δ* cells should lead to increased nuclear localization of Msn2. In agreement with this hypothesis, we observed that ~ 50% of cells grown under fermentation conditions displayed nuclear localization of Msn2 (S3 Fig.). In addition, the *ubr2Δ* strain exhibits the induction of genes such as *SUC2*, *HXK1* and *GAL1* that are typically derepressed upon glucose withdrawal, while the *rpn4Δ* strain exhibited reduced expression of these genes (Fig. 3). These data corroborate that cells with more proteasome activity exhibit a metabolic shift towards respiratory metabolism.

Since increased respiratory capacity was found under growth conditions that also promote efficient lifespan extension, we conclude that the ability to prematurely activate respiration might be an important component of proteasome-mediated effects on lifespan. Our results furthermore suggest that proteasome-mediated lifespan extension does not originate from imbalanced mitochondrial dynamics. We therefore speculated that increased or decreased proteasome activity might regulate signaling pathways that control metabolic adaptation.

### AMP-kinase/Snf1 signaling is involved in proteasome-mediated lifespan extension

**Figure 4:**
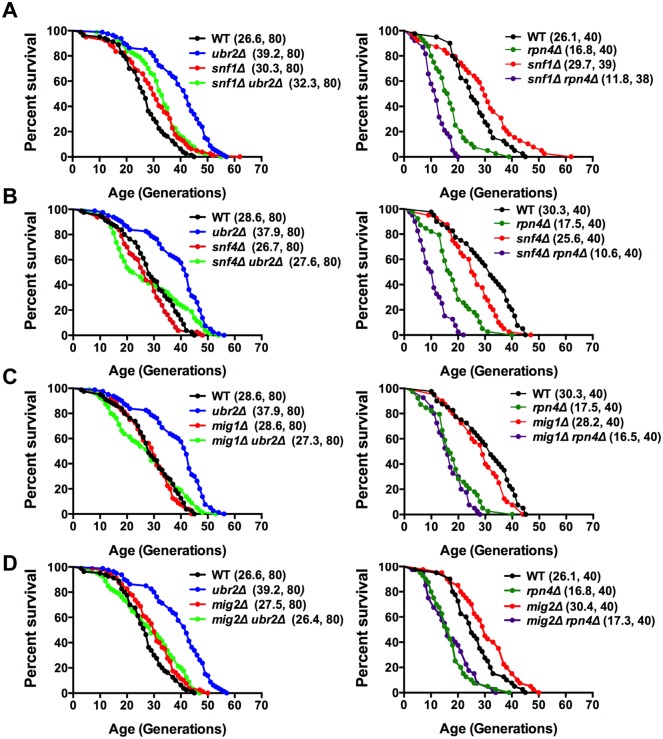
Snf1/AMPK signaling contributes to proteasome-mediated lifespan extension. (A) Loss of *SNF1* abrogates proteasome-mediated lifespan extension. Left panel: Survival curves of WT cells were compared to *ubr2Δ*, *snf1Δ*, and *ubr2Δ snf1Δ*. Right panel: Survival curves of WT cells were compared to *rpn4Δ*, *snf1Δ*, and *rpn4Δ snf1Δ*. (B) Loss of the Snf1 activator SNF4 abrogates proteasome-mediated lifespan extension. Left panel: Survival curves of WT cells were compared to *ubr2Δ*, *snf4Δ*, and *ubr2Δ snf4Δ*. Right panel: Survival curves of WT cells were compared to *rpn4Δ*, *snf4Δ*, and *rpn4Δ snf4Δ*. (C) Survival curves of cells deleted for *UBR2*, *MIG1* or *UBR2* and *MIG1* (left panel) or for *RPN4*, *MIG1* or *RPN4* and *MIG1* (right panel) are shown. (D) Survival curves of cells deleted for *UBR2*, *MIG2* or *UBR2* and *MIG2* (left panel) or for *RPN4*, *MIG2* or *RPN4* and *MIG2* (right panel) are presented.

The metabolic switch from fermentation to respiration is mediated by the AMPK signaling pathway [43]. Deregulation of that pathway in response to varying proteasome activity could be causative for the premature induction of respiration in cells with increased proteasome activity. This hypothesis predicts that cells deleted for sucrose non-fermenting 1 kinase (Snf1), the AMP-activated kinase in yeast, should attenuate proteasome-mediated lifespan extension. Loss of *SNF1* in cells with increased proteasome abundance indeed abrogates the positive impact on lifespan in *ubr2Δ* cells to a large extent (Fig. 4A, left panel). We furthermore observed intensive genetic interaction between proteasome mutants and cells devoid of *SNF1* (S4 Fig.). Interestingly, the *snf1Δrpn4Δ* double mutant exhibits a synthetic growth defect (S4B Fig.) that likely underlies its extremely short lifespan.

To obtain additional information on the impact of the Snf1 pathway on proteasome-mediated effects on lifespan, we tested whether other subunits of the heterotrimeric AMPK/Snf1 complex contribute to proteasome-mediated lifespan extension. The α-subunit Snf4 activates the catalytic Snf1 γ-subunit [43]. Although we observed a mild increase in lifespan upon deletion of *SNF1* in our background (Fig. 4A), we found that loss of *SNF4* led to a reduction in lifespan (Fig. 4B), consistent with mammalian reports that Snf1 activation has a beneficial impact on longevity [44]. The inconsistent RLS results observed for *snf1Δ* (Fig. 4A) and *snf4Δ* (Fig. 4B) cells might be caused by metabolic adaptations in the absence of *SNF1* as *snf1Δ* cells are unable to respire (S4B Fig.), in contrast to *snf4Δ cells* that do not exhibit growth defects on non-fermentable carbon sources. For proteasome-mediated lifespan extension, however, both proteins are required, as loss of *SNF4* almost completely abrogated lifespan extension by *ubr2Δ* (Fig. 4B).

Sip2 and Gal83 are Snf1 β-subunits that regulate the localization of the Snf1 complex [45]. Sip2 has recently been implicated in Snf1-mediated effects on lifespan [21]. We find, in agreement with Ashrafi et al. [21], that *sip2Δ* cells exhibit a slightly shortened lifespan, but loss of this subunit does not affect proteasome-mediated lifespan extension (S4A Fig., right panel). Similar to Sip2, deletion of *GAL83*, also shortens lifespan, but is not required for *ubr2Δ*-mediated lifespan extension (S4A Fig., left panel). A potential impact of these factors on proteasome-mediated lifespan extension could have been masked by redundancy among β-subunits [46].

### Snf1-regulated transcriptional repressors are required for proteasome-mediated lifespan extension

The AMPK/Snf1 regulated metabolic switch from fermentation to respiration is induced by a tightly controlled set of transcriptional regulators that repress or derepress genes in response to carbon source availability [47]. The function of many transcription factors is regulated by proteasomal degradation [31]. We therefore speculated that the positive impact of elevated proteasome levels on lifespan might be caused by increased degradation of a negative regulator of lifespan and component of the Snf1 signaling pathway. The primary downstream effectors of Snf1 are the transcriptional repressors Mig1 and Mig2 [48], homologs of the transcriptional repressors Wilm’s Tumor protein 1 (WT1) and Krüppel-like factor 16 (KLF16) in mammals [49]. In media with abundant glucose, Mig1 and Mig2 repress genes that promote oxidative metabolism and ATP generation through respiration. Upon glucose depletion or on media with non-fermentable carbon sources, Snf1 is activated and phosphorylates nuclear Mig1 and Mig2, which results in nuclear export causing derepression of genes required for the metabolic switch [50]. Since the Snf1-regulated transcription factors are required for modulating metabolism, we tested whether deletion of these genes has an impact on lifespan. Neither loss of *MIG1* nor loss of *MIG2 per se* had a strong impact on lifespan in the genetic background used here (Fig. 4C, D), nor was redundancy evident from the lifespan of a double deletion strain (S5A Fig.). Potential redundancy could also occur with a third related transcriptional repressor, Mig3, that is regulated by Snf1 [48], although the genes regulated by Mig3 do not overlap with genes regulated by Mig1 and Mig2 [48]. We found that *mig3Δ* cells exhibit a short lifespan and this phenotype was not affected by loss of Mig1, Mig2 or both (S5B, C Fig.). Although these data did not provide evidence that Mig1 or Mig2 have a substantial impact on longevity, we found that Mig1 is required for proteasome-mediated lifespan extension and is epistatic to compromised proteasome function found in cells lacking *RPN4* (Fig. 4C). Similar results were obtained with strains in the absence of Mig2 (Fig. 4D).

### Increased proteasome capacity results in enhanced turnover of the transcriptional repressor Mig1

**Figure 5:**
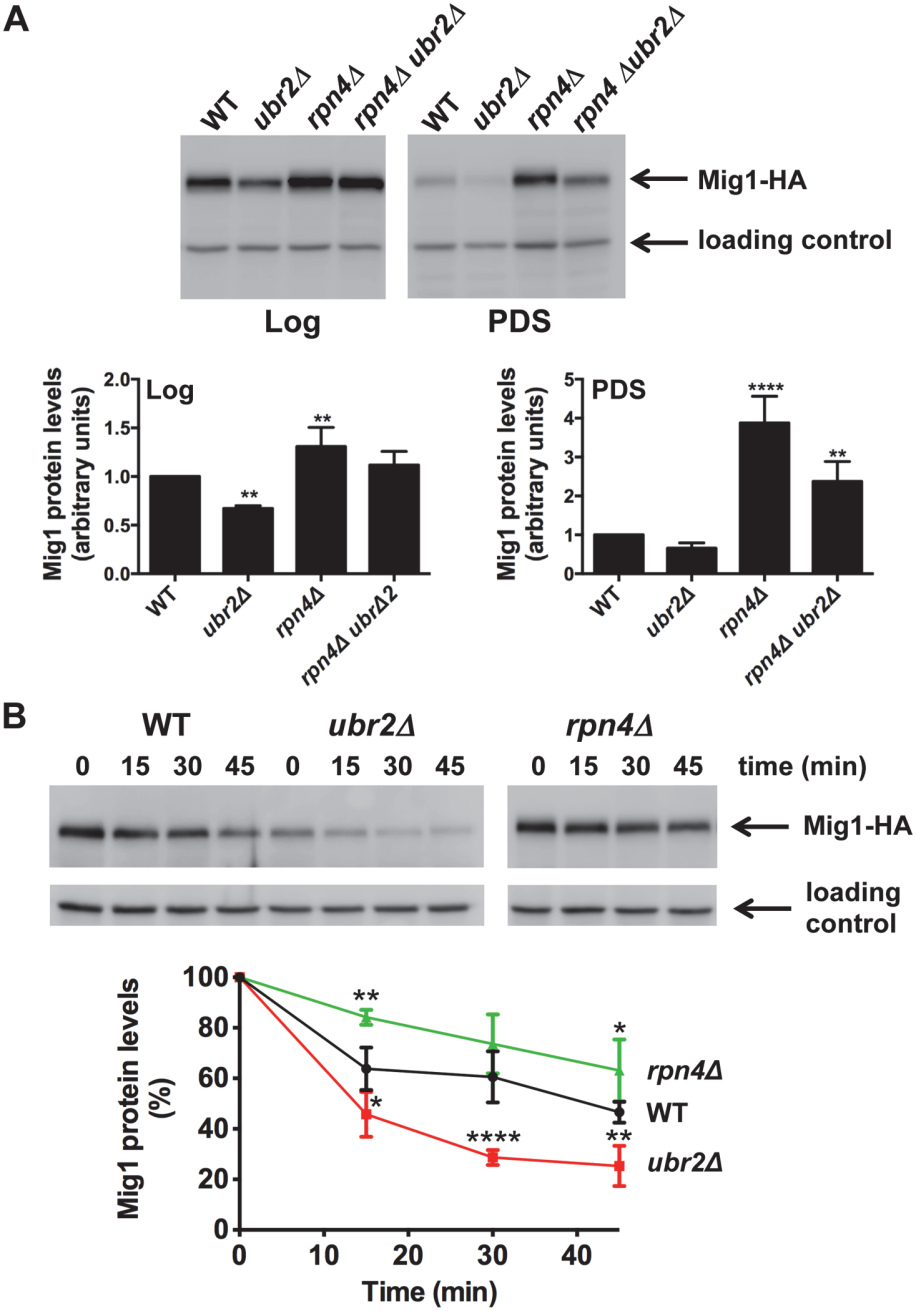
Proteasomes determine the abundance of the Snf1-regulated transcription factor Mig1. (A) Mig1 abundance correlates with proteasome capacity. Mig1 protein levels in normalized lysates were investigated via a C-terminal HA tag in cells with increased (*ubr2Δ*) or decreased (*rpn4Δ, rpn4Δ ubr2Δ*) proteasome activity under fermentative (log) or oxidative growth conditions (PDS). Detection of PGK1 was used as a loading control. The chemiluminescence signals of the immunoblot analysis were recorded in an Image Quant detection instrument (upper panels) and quantified using the Image Quant software (lower panels). The mean +- SD of four independent experiments is presented. *P*-values represent the statistical significance relative to WT expression and were assessed by an Ordinary one-way Annova using the GraphPad Prism software. *P*-values: * p < 0.05, ** p < 0.01, *** p < 0.001. (B) Mig1 is a proteasome target. The turnover of Mig1 on cells grown in galactose was determined in WT, *rpn4Δ*, and *ubr2Δ* cells after new synthesis was blocked with 200 μg/ml CHX. Mig1 levels were detected and quantified as in (A).

To gain mechanistic information on the functional interaction between the proteasome and AMPK/Snf1 signaling, we aimed to identify potential proteolytic targets within this signaling pathway. We analyzed the abundance of Snf1, Mig1, Mig2 and Hxk2, an important regulator of Mig1 and Snf1 [22, 51] in cells with increased or reduced proteasome abundance. We found that Snf1 abundance was unaltered in *ubr2Δ* or *rpn4Δ* cells both under fermentative (log) or oxidative growth conditions (PDS) (S6A Fig.). Hxk2 levels were also not affected by varying proteasome levels during logarithmic growth but were elevated after the shift to oxidative metabolism (PDS) in *rpn4Δ* cells (S6B Fig.). *ubr2Δ* cells on the other hand do not exhibit reduced Hxk2 levels. Thus, the increase observed in cells devoid of *RPN4* might not be a consequence of reduced proteasomal turnover. Mig1, however, was present at reduced levels in cells with increased proteasome activity (*ubr2Δ*) and at increased levels in cells with reduced proteasome activity (*rpn4Δ*) under both metabolic conditions (Fig. 5A). These data suggest that Mig1 is degraded by the proteasome. To unequivocally demonstrate that Mig1 is degraded by the proteasome we tested the degradation of Mig1 in cells with increased and decreased proteasome capacity using a cycloheximide (CHX) chase assay. We observed Mig1 turnover in WT cells (Fig. 5B). The rate of degradation was reduced in *rpn4Δ* cells and elevated in the *ubr2Δ* cells (Fig. 5B). Additionally, the proteasome-specific inhibitor MG132 reduced the turnover in WT and *ubr2Δ* cells (S7 Fig.). Thus, Mig1 abundance is regulated by the proteasome.

**Figure 6:**
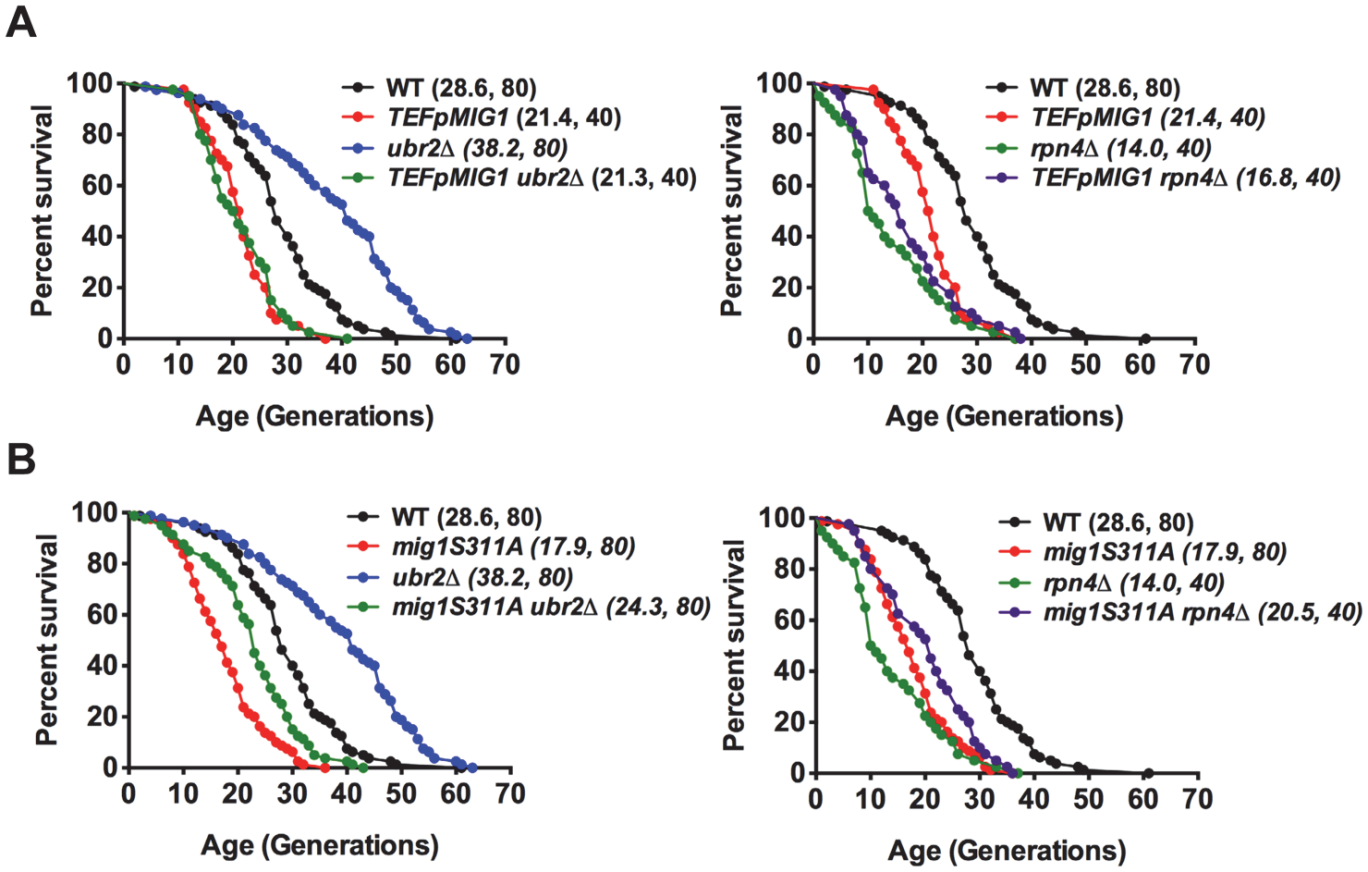
Overexpression of Mig1 as well as preventing Snf1-mediated Mig1 phosphorylation abrogates proteasome-mediated lifespan extension. Survival curves of WT cells were compared to cells that overexpress *MIG1* (*TEFpMIG1*) in the presence or absence of *UBR2* (A) or to cells that express a genomically integrated point mutant (*mig1S311A*) that prevents Snf1-mediated phosphorylation and nuclear export of Mig1 under repressive conditions in the presence or absence of *UBR2* (B). Mean lifespan and cell counts are shown in parenthesis. A statistical analysis of the data is summarized in S3 Table.

Our findings suggest that altered Mig1 levels in cells with increased or decreased proteasome activity might contribute to proteasome-mediated effects on lifespan. This hypothesis predicts that an artificial rise in Mig1 levels might compromise the lifespan extension observed in cells with increased proteasome activity. To test this we investigated lifespans in proteasome mutants where the endogenous Mig1 promoter has been exchanged against the constitutively overexpressing promoter of *TEF1*. In agreement with a previous study [52], overexpression of the gene resulted in a reduction in lifespan (Fig. 6A, *TEFpMIG1*) supporting the hypothesis that Mig1 is a negative regulator of lifespan. Interestingly, lifespan extension in cells with increased proteasome activity is abrogated upon *MIG1* overexpression. Thus, directed turnover of Mig1 (Fig. 5) is likely important for enhanced longevity through enhanced proteasome function.

### Increased proteasome activity correlates with incorrect Mig1 localization

The data presented in Fig. 6A demonstrate that Mig1 overexpression results in a shortened RLS. Consequently, the reduced turnover of Mig1 in cells with reduced proteasome activity might contribute to lifespan shortening in *rpn4Δ* cells (Fig. 4C, right panel). Surprisingly, however, the residual Mig1 in *ubr2Δ* cells is required for proteasome-mediated lifespan extension as complete loss of Mig1 abrogates lifespan extension (Fig. 4C, right panel). Two potential scenarios might explain these findings: 1) Loss of *MIG1* could result in a compensatory upregulation of a negative regulator of lifespan. As a potential candidate we investigated the level of Hxk2, but found no evidence for increased abundance (S6C Fig.). 2) Alternatively, in addition to its function as a transcriptional repressor, Mig1 could have a second function that positively contributes to longevity.

**Figure 7:**
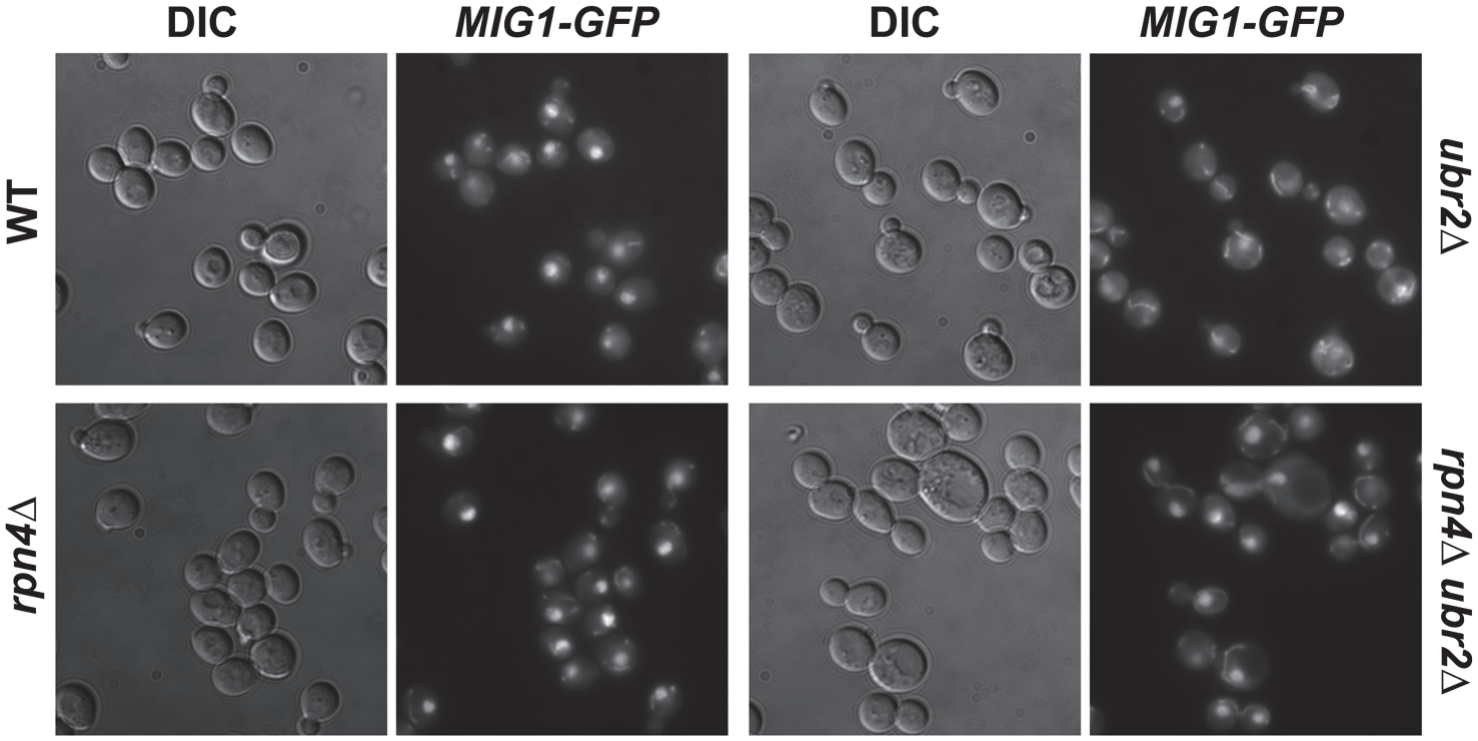
Mig1 is mislocalized in in cells with increased proteasome capacity. Reduced nuclear localization of Mig1 in exponentially growing *ubr2Δ* cells. Mig1 localization was analyzed via a C-terminally fused GFP tag using live cell fluorescence imaging of the strains indicated. Projected sequential Z-stacks fluorescence images are presented. DIC: differential interference contrast.

**Figure 8:**
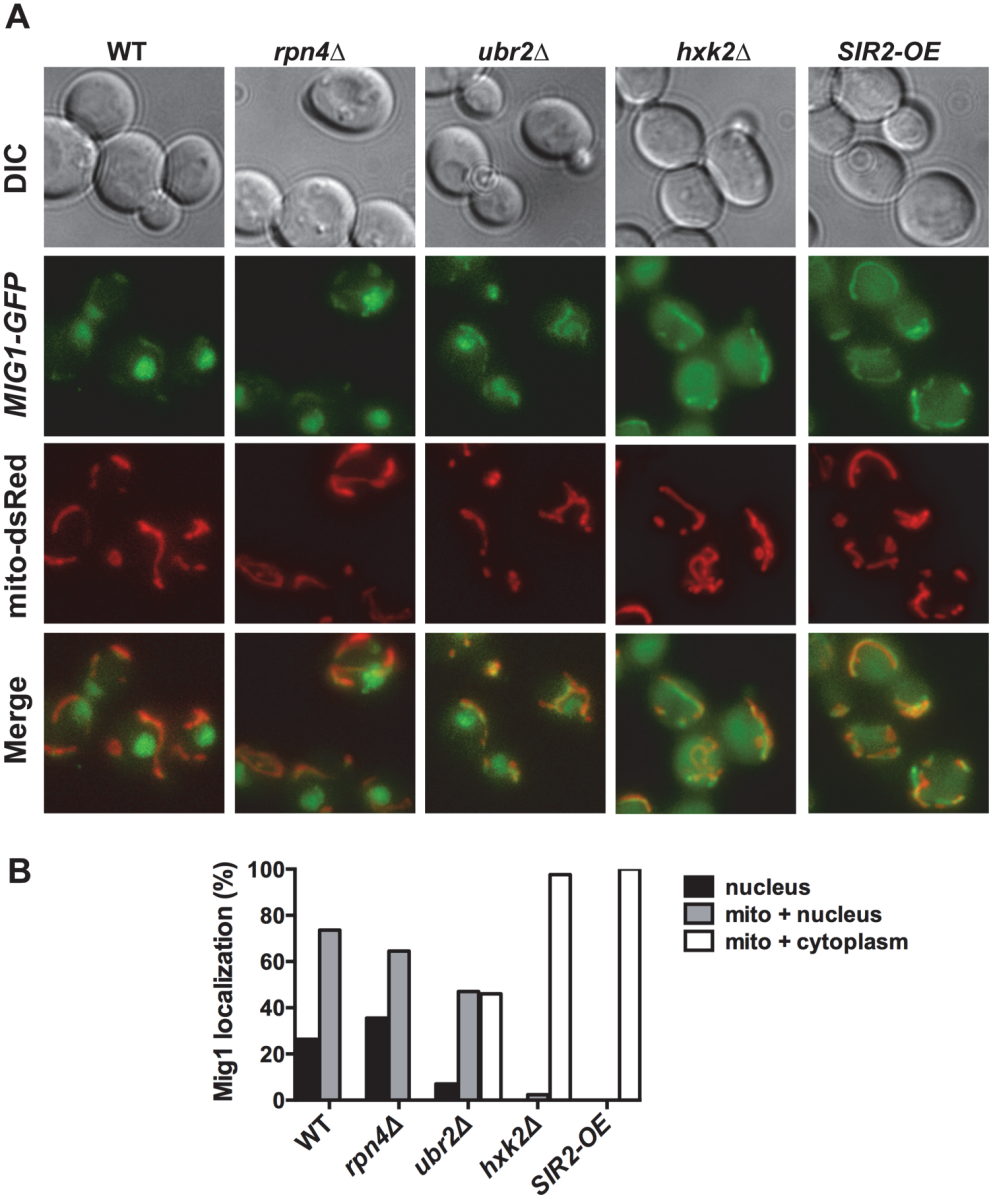
Mig1 co-localizes with the mitochondria in cells with increased proteasome abundance, in the absence of *HXK2* and upon overexpression of *SIR2*. (A) The strains indicated carrying Mig1 with a C-terminally fused GFP tag were co-transfected with the plasmid mito-dsRED, carrying a red fluorescing mitochondrial marker. Co-localization of Mig1 with the mitochondria is visualized via merging of the Mig1 and the mitochondrial signals in living cells during early logarithmic growth. Projected sequential Z-stacks fluorescence images are presented. DIC: differential interference contrast. Mig1 localization of ~300 cells for each strain was analyzed and presented in (B).

Under derepressed conditions, Snf1-mediated phosphorylation of Mig1 triggers its nuclear export and results in derepression of its target genes [51]. Mig1 export from the nucleus into the cytoplasm has also been reported in aged mother cells [21], suggesting that the nuclear export of Mig1 might be relevant for replicative lifespan. We therefore constructed a genomically integrated Mig1 point mutant (*mig1S311A*) that has been previously demonstrated to prevent Mig1 phosphorylation and nuclear export in response to Snf1 activation [51]. Interestingly, this mutant is short-lived and strongly reduces lifespan extension in cells with increased proteasome activity (Fig. 6B) demonstrating that Mig1 export is a crucial mechanism that affects longevity in cells with increased proteasome activity. We therefore considered the possibility that cytoplasmic Mig1 assumes a function that is distinct from its nuclear role as a transcriptional repressor during respiratory growth and analyzed its localization in proteasome mutants. While all WT cells displayed a predominantly nuclear localization of Mig1, ~50% of *ubr2Δ cells* exhibited a cytoplasmic distribution of Mig1 (Figs. 7 and 8). Additionally, we observed that Mig1 was recruited to the mitochondria in cells with increased proteasome capacity (Figs. 7 and 8, merged panels). Since a recent report demonstrated that Mig2 re-localizes to the mitochondria after Snf1 activation and the induction of respiration [53], our data suggest that both homologs can be re-routed to the mitochondria.

Disruption of *MIG2* results in a hyperfragmented mitochondrial network [53]. We therefore anticipated that if the recruitment of Mig1 to the mitochondria has functional consequences similar to mitochondrial recruitment of Mig2 after the diauxic shift, we might observe changes in mitochondrial morphology. Similar to loss of *MIG2*, deletion of *MIG1* causes hyperfragmentation of the mitochondrial network (S8 Fig.). This could indicate that Mig1 impacts mitochondrial morphology or respiratory capacity.

### The proteasome, Sir2 and Hxk2 form an interconnected aging network

To further explore the impact of Mig1 on yeast longevity, we measured the lifespan of cells deleted for *MIG1* in two additional strains that confer robust lifespan extension in yeast: *SIR2-OE* and *hxk2Δ*. Hxk2 is a protein with two functions: it catalyzes the first step in glycolysis, but also is involved in regulating AMPK/Snf1 signaling [54]. Deletion of the gene results in increased respiration, a transcriptional profile similar to cells under caloric restriction and robustly extends replicative and chronological lifespan in yeast [2, 22, 55]. Hxk2 also directly regulates Mig1 and loss of Hxk2 results in Mig1 inactivation [51]. As a consequence, Mig1 fails to be imported into the nucleus in the absence of *HXK2* (Fig. 8B) [51]. Since *HXK2* is an upstream activator of *MIG1* we expected that loss of *MIG1* should not affect the long lifespan of cells in the absence of *HXK2*, which we confirmed (Fig. 8A).

**Figure 9:**
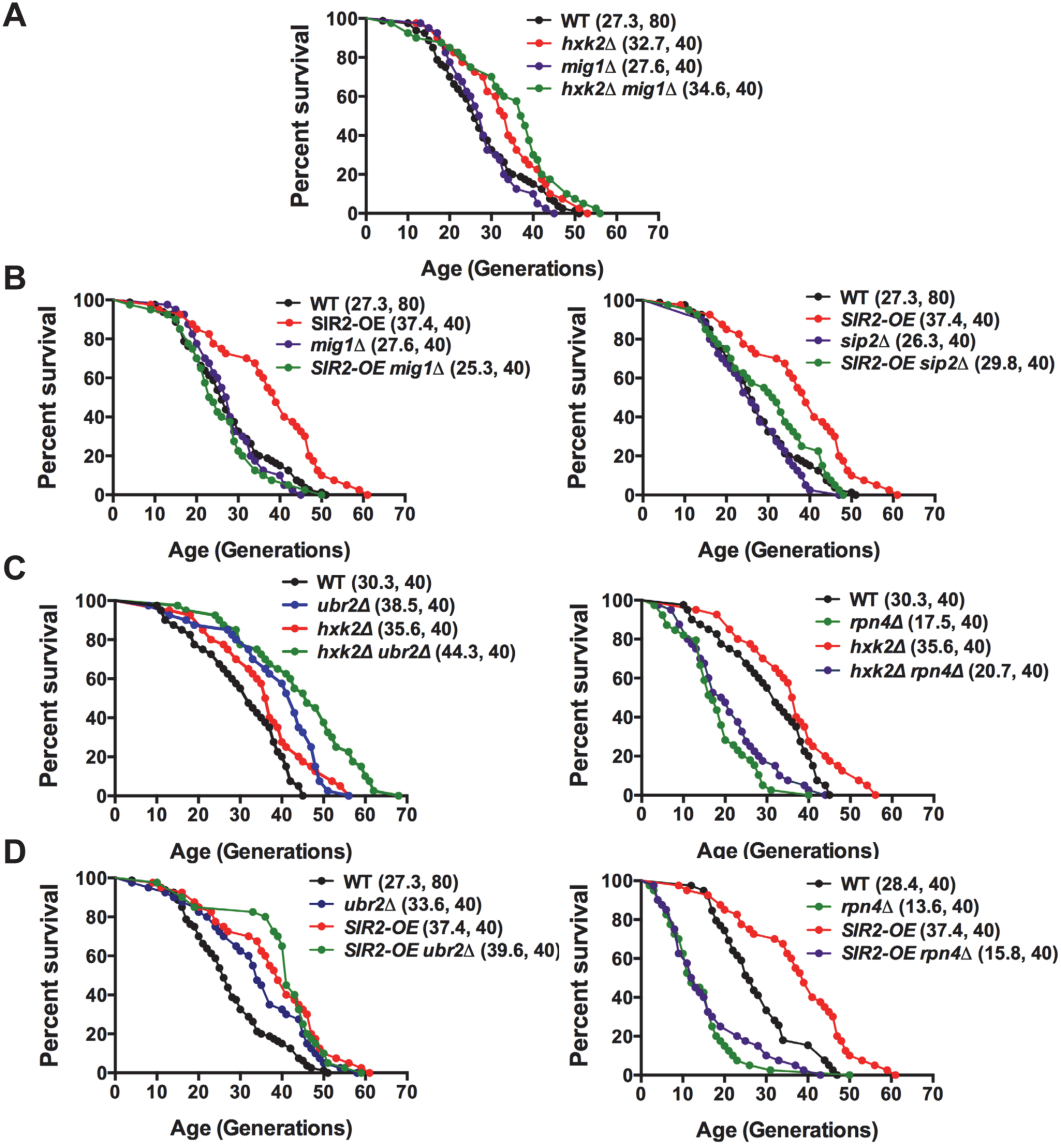
Mapping genetic interactions between different aging pathways. (A) Mig1 is not required for *hxk2Δ* mediated lifespan extension. Survival curves of *hxk2Δ*, *mig1Δ or mig1Δ hxk2Δ* cells. (B) Incorrect AMPK/Snf1 signaling impairs SIR2-OE mediated lifespan extension. Survival curves of *SIR2-OE*, *mig1Δ or mig1Δ SIR2-OE* cells. (C) Decreased proteasome abundance abrogates lifespan extension in cells deleted for *HXK2*. Survival curves of cells with increased (*ubr2Δ*, left panel) or reduced proteasome capacity (*rpn4Δ, right* panel) co-deleted for *HXK2*. (D) Decreased proteasome abundance abrogates lifespan extension in cells overexpressing *SIR2*. Survival curves of *SIR2-OE* cells with increased (*ubr2Δ*, left panel) or reduced proteasome capacity (*rpn4Δ*, right panel). Mean lifespan and cell counts are shown in parenthesis. A statistical analysis of the data is summarized in S3 Table.

Overexpression of *SIR2* confers increased longevity [56]. While there is no information available in yeast, the mammalian ortholog of Sir2, SIRT1 has been linked previously to AMPK function. SIRT1 regulates the activity of LKB1 kinase, an activator of mammalian AMPK [57]. On the other hand, AMPK activation results in increased NAD^+^ levels, which in turn activates the activity of SIRT1 [58]. Thus, SIRT1 and AMPK are functionally linked through a positive feedback loop in mammalian cells [59]. We therefore speculated that loss of the AMPK/Snf1 target *MIG1* might impact *SIR2-OE* longevity in yeast. Interestingly, deletion of *MIG1* in a strain overexpressing *SIR2* results in a growth defect in the presence of glucose or non-fermentable carbon sources (S6 Fig.). The cells also are exquisitely sensitive to oxidative stress induced by the addition of cadmium chloride to the medium (S6 Fig.). Additionally, we observed that loss of *MIG1* abrogates Sir2-mediated RLS extension (Fig. 9B, left panel). These results are similar to the loss of lifespan extension mediated by proteasome overexpression in the absence of *MIG1* (Fig. 4C, left panel). Furthermore, deletion of the Snf1 β-subunit Sip2 reduces *SIR2-OE*-mediated lifespan extension (Fig. 9B, left panel). To test whether Sir2 has an impact on Mig1, we investigated the localization of Mig1 in cells that overexpress *SIR2*. Similar to cells lacking *HXK2* and partially in *ubr2Δ* cells, Mig1 import into the nucleus under repressive conditions is impaired (Fig. 8). These observations indicate an interaction between AMPK/Snf1 signaling and Sir2-mediated effects on lifespan and suggest that Sir2 negatively regulates Mig1.

**Figure 10:**
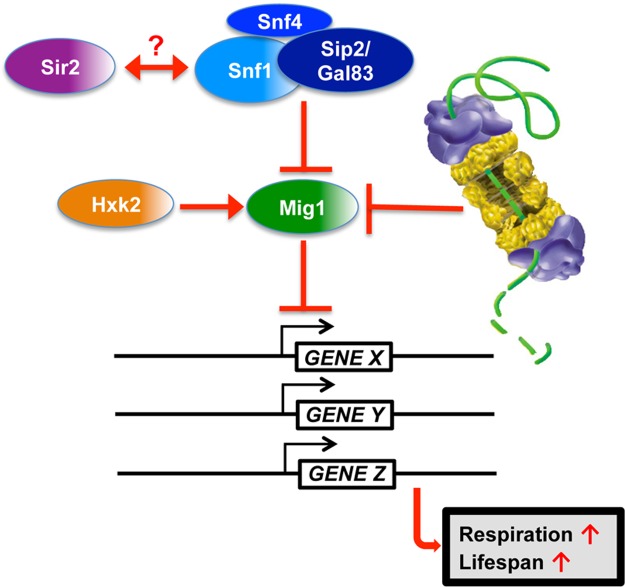
Model for Mig1 regulation by Snf1, Hxk2, the proteasome and Sir2.

Having established that AMPK/Snf1 signaling affects *SIR2-OE*-mediated lifespan extension and that increased proteasome activity results in deregulation of the kinase signaling pathway, we hypothesized that potentially varying proteasome levels could also affect the impact of Sir2 or Hxk2 on lifespan. We therefore determined whether changes in proteasome activity alter *SIR2-OE* and *hxk2Δ*-mediated lifespan extension. In both strains, increased proteasome activity led to a slightly additive affect (Fig. 9 C, D, left panels). Interestingly, reduced proteasome activity completely blocked lifespan extension for both strains (Fig. 9 C, D, right panels) potentially a consequence of the increased Mig1 levels in *rpn4Δ* cells (Fig. 4C) as *MIG1* overexpression shortens lifespan [52]. These findings point to an intricate link between AMPK/Snf1 signaling, Hxk2, Sir2 and the proteasome that impacts lifespan at least partially through modulation of Mig1 activity as depicted in the model presented in Fig. 10.

## Discussion

The activity of the proteasome is required for cellular fitness at many levels. It is a crucial component of the cellular network that maintains protein homeostasis and proteasome-mediated degradation is required for the regulation of a multitude of pathways through the targeted and irreversible elimination of individual components. Many studies demonstrate that proteasomal activity declines in aging cells [30, 60] and we and others have shown that elevated proteasome capacity has a beneficial effect on lifespan [24, 25, 26, 30]. We reported that increased proteasome capacity ameliorates proteotoxic stress [24] and a recent study demonstrated that elevated proteasome activity reduces the aggregation of endogenous misfolding proteins in aged mother cell [30]. These findings raise the possibility that the beneficial effect of enhanced proteasomal degradation in aging cells might largely be attributed to improved protein homeostasis. Here we show that increased proteasome activity also results in altered metabolic signaling, mediated by deregulation of the AMPK/Snf1 pathway and that this activity is required for the positive impact of enhanced proteasome function in aging cells. We demonstrate that proteasomes participate in the regulation of AMPK/Snf1 signaling through degradation of the transcriptional repressor Mig1. Cells with increased proteasome activity exhibit reduced Mig1 levels, increased expression of genes required for the induction of respiratory metabolism, enhanced oxidative stress response and elevated respiratory capacity. The data support a model in which the pro-longevity effect of increased proteasome function in aging cells partially originates from a premature induction of respiration resulting in a mitohormetic response.

Previous reports support a complex functional link between the proteasome and mitochondria. Compromised mitochondrial activity results in decreased proteasomal degradation [61, 62] and dissociation of the proteasome holocomplex [63, 64] most likely caused by oxidative modification of proteasome subunits. Conversely, treating cells or model organisms with proteasome inhibitors impairs mitochondrial activity resulting in increased ROS levels and enhanced mitophagy [65, 66]. Additionally, a reduction in proteasome assembly in response to deletion of the assembly factor *UMP1* causes enhanced instability of mitochondrial DNA [67]. The results presented here provide additional information on the mutual interaction between mitochondria and proteasomes, by demonstrating that proteasomal activity is required for correct execution of AMPK/Snf1 signaling and thus for regulation of mitochondria biogenesis.

The AMPK/Snf1 signaling pathway is highly conserved, representing a key sensor of the cellular energy level that regulates metabolic adaptation and mitochondrial biogenesis. Although many studies implicate the AMPK/Snf1 pathway in modulating the aging process, its precise role in aging cells is unclear [44]. AMPK/Snf1 is activated in aging cells [68] or after the administration of CR mimetic drugs such as rapamycin or resveratrol [44]. However, overexpression of yeast *SNF1* shortens lifespan [21, 22] and overexpression of the AMPK activator LKB1 can induce senescence in certain eukaryotic systems [69]. Thus, the activity of Snf1 needs to be tightly regulated. Our data reveal a novel mechanism that contributes to the fidelity of AMPK/Snf1 signaling: proteasomal degradation of AMPK/Snf1-regulated transcription factors.

Mig1, Mig2 and Hxk2 are crucial downstream components of the AMPK/Snf1 signaling pathway in yeast. Several studies link these factors to longevity regulation for both replicative and chronological aging paradigms in yeast. A network biology approach predicted and confirmed that Snf1, Hxk2 and Mig1 are modulators of chronological lifespan in yeast [22] and these genes also contribute to replicative aging [52]. These findings are in agreement with a recent study that demonstrated that mitochondrial function is crucial for both yeast aging paradigms [70]. While both deletion and overexpression of *SNF1* shortens lifespan [22], deletion of *HXK2* results in a robust lifespan extension [2, 71]. Transcriptional and metabolomic analyses indicate that cells devoid of *HXK2* likely exhibit a CR profile [2, 50] and Hxk2 was found to be a key integrator of glucose sensing and metabolic adaptation in aging cells [22]. Hxk2 has also an enzymatic function in glycolysis as it phosphorylates glucose [72] and impaired initiation of glycolysis in cells devoid of *HKX2* might contribute to *hxk2Δ*-mediated lifespan extension. *MIG1* overexpression accelerates replicative and chronological aging and loss of *MIG2* enhances RLS [52]. Our data indicate that Mig1 and Mig2 have a redundant function in regulating lifespan. While deletion of either gene had a marginal impact of lifespan, co-deletion conferred robust extension. In summary, the three AMPK/Snf1 regulated transcriptional repressors, Hxk2, Mig1, and Mig2 are negative regulators of lifespan.

Our data furthermore demonstrate that Mig1 is (1) a proteasome target, (2) required for proteasome-mediated lifespan extension and (3) is incorrectly processed under repressive conditions in cells with increased proteasome abundance. Moreover, increased proteasome activity leads to incorrect localization of the transcriptional repressor under repressive conditions. These findings correlate with increased respiration, elevated oxidative stress response and increased expression of AMPK/Snf1 target genes. Thus, our data are consistent with a model in which increased proteasomal degradation of Mig1 leads to a partial inactivation of Mig1 and in consequence to a premature activation of the AMPK/Snf1 pathway. A link between Mig1 and the ubiquitin/proteasome system is further supported by a recent report demonstrating that *MIG1* deletion rescues the lifespan defect of a mutant anaphase-promoting complex (APC), an E3 ubiquitin ligase complex that is required for optimal lifespan [52].

Surprisingly, however, deletion of *MIG1* abrogated the lifespan extension in cells with increased proteasome activity, indicating that Mig1 is required for proteasome-mediated lifespan extension. We also observed that Mig1 exhibited reduced nuclear localization and relocalized to the mitochondria in cells with increased proteasome activity under repressive conditions and a mutant (*mig1S311A*) that prevents nuclear export of Mig1 abrogates proteasome-mediated lifespan extension. Two potential scenarios could explain the impaired lifespan extension in the absence of *MIG1*: a) loss of *MIG1* induces increased expression of a second negative regulator of lifespan that overrides the pro-longevity effect of increased proteasome activity or b) the protein has a dual role as both a negative and positive regulator of lifespan. Under repressive conditions Mig1 functions as a transcriptional repressor to prevent premature activation of respiration [22, 50]. Under starvation conditions, however, the protein relocalizes to the mitochondria and promotes mitochondrial function by an unknown mechanism. Interestingly, a previous study showed that the Mig1 partner, Mig2 also exits the nucleus and is recruited to the mitochondria upon Snf1 activation by glucose depletion [53]. Although the functional consequences of Mig2 recruitment to the mitochondria are not fully understood, the mitochondria fragment in the absence of *MIG2* upon glucose depletion and deletion of Mig2 also rescues the hyperfused mitochondria phenotype of cells deleted for the mitochondrial fission protein Dnm1 [53]. Thus, Mig2 appears to antagonize mitochondrial fission during respiratory growth. Mig1 has not been investigated in that context. Our analysis shows, however, that like Mig2, Mig1 also impacts mitochondrial dynamics as *mig1Δ* cells exhibit a high percentage of fragmented mitochondria. Whether the incorrect mitochondrial morphology is a primary consequence of Mig1 preventing mitochondrial fission or whether it is a secondary effect in response to altered mitochondrial function is currently not known. A dual mitochondria-related function of Mig1/2, however, would point to a scenario where these proteins are involved in nuclear-mitochondrial communication [73].

The data presented also indicate a mutual link between AMPK signaling and lifespan extension mediated by elevated S*IR2* expression as both *MIG1* deletion as well as deletion of the Snf1 β-subunit *SIP2* abrogated the beneficial impact of *SIR2* overexpression on longevity. Sir2 is a protein-deacetylase with histones as primary targets and its positive impact on yeast lifespan was largely attributed to a reduction in the formation of extrachromosomal rRNA circles (ERCs) that impede lifespan [56]. Our data, however, link *SIR2*-mediated lifespan extension in yeast to AMPK/Snf1 signaling and are consistent with findings in mammalian cells demonstrating that altered NAD/NADH levels in cells with compromised AMPK signaling modulate Sir2 since NAD is a substrate [74]. Our findings also indicate that Sir2 activity might impact metabolic regulation, as Mig1 localization was affected in cells overexpressing *SIR2*. Similar to cells with increased proteasome function, the nuclear localization of Mig1 was abrogated in this strain, suggesting that Sir2 is involved in negative regulation of Mig1, potentially through deacetylation of non-histone proteins within the AMPK/Snf1 signaling pathway.

In this study, we have identified proteasomes and Sir2 as negative regulators of Mig1. In both cases enhanced activity has a pro-longevity effect. In line with these findings is the observation that deletion of a positive regulator of Mig1, *HXK2*, also induces robust lifespan extension. Additionally, we observed that *MIG1* deletion abrogates both proteasome and Sir2-mediated lifespan extension, while this genetic intervention had no effect on lifespan extension in the absence of *HXK2*, consistent with Hxk2 being an upstream activator of Mig1. We furthermore observed that reduced proteasome activity abrogates lifespan extension in cells with increased Sir2 levels or deleted for *HXK2*. The congruent genetic and functional interactions are indicative of synergy between AMPK/Snf1 signaling, proteasome-mediated degradation and Sir2-dependent deacetylation in aging cells and argue for a model in which these pathways form an interconnected network that impinges on Mig1 to regulate respiration and lifespan (Fig. 10).

## Materials and Methods

### Strains and media

All strains are isogenic to BY4741 or BY4742 and are S288C derivatives [75]. The genotypes are listed in S1 Table. Complete gene deletion, promoter exchange or tag integration was performed at the genomic locus by homologous recombination using standard genetic techniques. Unless otherwise noted strains were grown in YPD medium (1% yeast extract, 2% bactopeptone, 2% glucose). Experiments with alternate carbon sources were done with 2% galactose, 2% raffinose or 3% glycerol replacing glucose. For logarithmic cultures cells were grown to O.D. < 0.5 and post-diauxic shift cultures were harvested after overnight incubation. *mig1S311A* strains were constructed using the 50:50 method for marker-free, seamless genome editing as described previously [76]. Briefly, the *URA3* cassette from vector pRS416 was amplified via polymerase chain reaction (PCR) using two specific primers, a 50:50 primer and a standard reverse primer. The 50:50 primers were designed as follows: (5′ to 3′) 50 nucleotides (nt) directly upstream of *MIG1S311* and 50 nt directly downstream of *MIG1S311* and a sequence for priming the *URA3* locus.

YY220:GAAGTCTCAGAGCACAAACTCAGAGTTCCGTACAGTTGAAGAGACCAAGTGCAGTTTTAAGTTTGAACGACTTGTTGGTTGGCCAAAGAAATACCAACGAATCggcttaactatgcggcatcag

The reverse primer contained 50 nt directly downstream of *MIG1S311* and a sequence for priming *URA3* YY221:GATTCGTTGGTATTTCTTTGGCCAACCAACAAGTCGTTCAAACTTAAAACcgtttacaatttcctgatgcgg.

A standard homologous recombination transformation method with this PCR cassette was performed in BY4742, yMS1773 and yMS1776 strains. Correct transformants were selected on medium in the absence of uracil, identified by genomic PCR using primers specific to both sides of the integrated *URA3* cassette and transferred on 5-fluoro-orotic acid (5-FOA) medium to remove *URA3* cassette. The correct mutation was finally confirmed by DNA sequencing.

### Replicative lifespan analysis

Replicative lifespan assays were carried out as described previously [24]. Unless otherwise noted, all lifespan experiments were performed on YPD plates with 2% glucose. Lifespan curves in Figs. 1A, C, D, and 7D were compiled from multiple assays containing experimentally matched WT and single mutant controls.

### Analysis of respiratory capacity

The oxygen consumption of WT, *ubr2Δ, rpn4Δ*, and *ubr2Δ rpn4Δ* cells was measured at 30°C using an Oroboros instrument (Oroboros, Graz, Austria). Respiratory rates JO were determined from the slope of a plot of O_2_ concentration versus time. For all assays 2 ml of a growing cell suspension were used. Respiration assays of growing cells were performed in YEP + 2% galactose or 2% lactate when applicable triethyltin bromide, TET (Sigma) was added at 0.2 mM and carbonyl cyanide *m*-chlorophenyl hydrazone, CCCP (Sigma) at 10 μM. RSV, respiratory state value is defined as (JO_2basal_–JO_2TET_)/ (JO_2CCCP_–JO_2TET_). RSV represents the percentage of stimulation of oxidative phosphorylation compared to the basal respiration capacity [41].

### Live cell fluorescence microscopy

Mitochondria-targeted plasmids Cox4-GFP (pOK29) [77] or plasmid mito-dsRed [78] were introduced into the strains indicated via transformation. The cells were grown in SD medium including 2% glucose and imaged at the early logarithmic growth of O.D._660nm_ 0.4–0.5. The localization of Msn2 and Mig1 during logarithmic growth in SD medium including 4% glucose was investigated via a genomic integration of GFP at the C-terminus of both proteins followed by live cell fluorescence imaging. Live cell fluorescence was monitored using a fluorescence microscope (Olympus BX61) at the Albert Einstein Imaging Facility with a 100X NA 1.4 objective (PlanApo). Fluorescence or differential interference contrast (DIC) images were captured with a cooled CCD camera (Sensicam QE) using IPlab 4.0 software. Images were identically processed using ImageJ software 1.42q. For excitation of mtGFP, a 470–495 nm band pass filter was used and a 530–582 nm filter was used for mtRed. Emitted light was detected with a 510–550 nm long pass filter for mtGFP and a 600–665 nm filter for mtRed (filter set Olympus). For visualizing mitochondria 20 Z-section images were captured with a 0.2 γm distance between two neighboring sections. Projected sequential Z-stack images were created in ImageJ 1.42q. Overlays between Mig1 and mitochondrial signals were created in Adobe Photoshop CS5.1.

### Quantitative Real-Time PCR analysis

Strains were grown to mid-log phase (O.D._600nm_ = 0.5) in 2% glucose medium, then switched in 2% galactose medium and inoculated for 4 hours. Total RNA was prepared using a standard phenol-chloroform extraction method. 1 μg RNA was treated with DNase I (Life Technilogies), followed by reverse transcription using the High Capacity cDNA kit (Applied Biosystems). cDNA from 1 μg RNA was subjected to qRT-PCR using the LightCycler 480 SYBR Green I Master (Roche) in the LightCycler 480 Instrument (Roche). The reactions were performed in 45 cycles with 95°C for 10 sec, 55°C for 20 sec and 72°C for 30 sec after an initial activation at 95°C for 5 min. Negative controls were run simultaneously for each reaction. Data were analyzed using the LightCycler 480 software. To compare the relative mRNA expression between the individual genes and the reference gene *ACT1*, the comparative threshold cycle (CT) method was used. The amount of target, relative to the reference gene as described in the Fig. legends, is given by 2^-ΔΔCT^. All reactions were performed in triplicates. Error bars indicate the standard error of the mean (± SEM) of three independent experiments. One-way ANOVA tests were performed between strain transcript level averages to assess significance using the GraphPad Prism 6 software. Primers are listed in S2 Table.

### Gel electrophoresis and immunoblotting

Cells from WT and mutant strains were harvested in the respective growth phases at O.D._660nm_:1 for logarithmic cultures and at O.D._660nm_:>8 for post-diauxic cultures. Cells were disrupted by alkaline lysis as described previously [79] with modifications. TE buffer (10mM Tris pH7.5, 1mM EDTA) was used instead of water. Protein concentration was determined by a Bradford protein assay (BioRad). Equal amounts of proteins were loaded on 12% SDS-polyacrylamide gels and subjected to electrophoresis. The gels were blotted onto PVDF membranes. The membranes were probed with anti-HA 12C5 (Roche) antibodies to detect Hxk2, Mig1 and Fzo1. Anti-Flag (Stratagene) was used to visualize Snf1. Anti-phosphoglycerate kinase (Pgk1) antibodies (Invitrogen) were used as a loading control. Protein bands were detected via enhanced chemiluminescence using an ImageQuant LAS4000 mini system (GE Healthcare). The protein intensity was quantified using the ImageQuant TL software (GE Healthcare).

### Cycloheximide chase assay

Overnight cultures of WT, *ubr2Δ*, and *rpn4Δ* cells expressing *MIG1–3HA* were diluted to O.D_660nm_: 0.5 and incubated for 2h in YPD. Cells were washed, transferred into YPGal and grown for 4h prior to the addition of 200 μg/ml CHX. For proteasome inhibition, *PDR5*, a gene that encodes a drug efflux pump [80], was deleted in WT, *ubr2Δ*, or *rpn4Δ* strains. Cells were grown in YPGal for 1.5h before 100 μM MG132 (CalBiochem) or DMSO were added and incubated for another 1.5h in the presence of the inhibitor prior to the addition of 200 μg/ml CHX. At the time indicated aliquots were harvested and frozen in liquid nitrogen. After alkaline lysis [59] and measuring the total protein concentration with a Bradford reagent (Pierce), 20 μg were subjected to SDS-PAGE and immuno-detection using an anti-HA polyclonal antibody (Roche). anti-Pgk1 antibodies (Invitrogen) were used as a loading control. Signals were visualized using ECL chemiluminescence in an ImageQuant LAS 4000 imager (GE Healthcare) and quantified using the ImageQuant software package. Statistical analysis was performed with GraphPad Prism 6 using two-way ANOVA with grouped analysis and is based on three independent experiments.

## Supporting Information

S1 Table Strains used in this study. (DOCX)

S2 Table Primers used for qRT-PCR. (DOCX)

S3 Table Statistical analysis of the RLS experiments presented. *P*-value matrices for Figs. 1, 4, 6, and 9 and S4A and S5 Figs. *P*-values were assessed by a Wilcoxon test. Ranksum *p*-values: * *p*< 0.05, ** *p* < 0.01, *** *p* < 0.001, ns = not significant L: Left panel; R: Right panel.
(DOCX)

S1 Fig Increased proteasome abundance results in hyper-fragmented mitochondria in cells grown in media with galactose as carbon source. To visualize mitochondria GFP was tagged with a mitochondrial import signal and introduced in cells with increased (*ubr2Δ*), decreased (*rpn4Δ*) proteasome capacity and in cells deleted for *FZO1*. Projected sequential Z-stacks fluorescence images are presented. DIC: differential interference contrast.
(TIF)

S2 Fig Proteasome abundance impacts Fzo1 levels, but does not change the respiratory capacity in cells grown on non-fermentable carbon sources. (A) The levels of the mitochondrial fusion protein Fzo1 correlate with proteasome abundance. Fzo1 protein levels were investigated in 20 μg normalized lysates from the strains indicated via a C-terminal HA tag utilizing HA specific antibodies. Detection of PGK1 was used as a loading control. The chemiluminescence signals of the immunoblot analysis were recorded in an Image Quant detection instrument (upper panels) and quantified using the Image Quant software (lower panels). The mean +- SD of three independent experiments is presented. *P*-values represent the statistical significance relative to WT expression and were assessed by an Ordinary one-way Annova using the GraphPad Prism software. *P*-values: * p < 0.05, ** p < 0.01, *** p < 0.001. (B) Mitochondrial activity is not affected by proteasome abundance in media containing the non-fermentable carbon source lactate. Experimental procedure as described in Fig. 2A.
(TIF)

S3 Fig Increased nuclear localization of the stress-responsive transcription factor Msn2 in cells with increased proteasome abundance. Upper panels: The localization of Msn2 in WT or *ubr2Δ* cells was visualized via a C-terminal GFP tag with live cell fluorescence microscopy. Lower panel: Quantification of cells with nuclear Msn2 localization was performed via visual inspection of ~ 300 cells in WT, *rpn4Δ*, *ubr2Δ, or rpn4Δ ubr2Δ* cells.
(TIF)

S4 Fig Phenotypic and lifespan analysis of proteasome mutants in the presence or absence of *SNF1, SIP2* or *GAL83*. (A) Cells with increased proteasome abundance rescue the short lifespan of cells deleted for the Snf1 β-subunits *GAL83* and *SIP2*. Survival curves of WT cells were compared to *ubr2Δ*, *gal83Δ*, and *ubr2Δ gal83Δ*. Right panel: Survival curves of WT cells were compared to *ubr2Δ*, *sip2Δ*, and *ubr2Δ sip2Δ*. Mean lifespan and cell counts are shown in parenthesis. A statistical analysis of the data is summarized in S3 Table. (B) Phenotypic analysis of proteasome mutants deleted for *SNF1*: 5-fold serial dilutions of the strains indicated were spotted on YPD and incubated at 30°C or 37°C (upper panels or were spotted on media with acetate as the sole carbon source or on YPD supplemented with cadmium chloride.
(TIF)

S5 Fig The impact of Mig1 related transcriptional repressors on lifespan. Survival curves of WT cells were compared to (A) *mig1Δ*, *mig2Δ*, and *mig1Δ mig2Δ;* (B) *mig1Δ*, *mig3Δ*, and *mig1Δ mig3Δ* and (C) *mig1Δ*, *mig2Δ, mig3Δ, mig1Δ mig2Δ* and *mig1Δ mig2Δ mig3Δ*. Mean lifespan and cell counts are shown in parenthesis. A statistical analysis of the data is summarized in S3 Table.
(TIF)

S6 Fig Altered proteasome abundance does not affect the levels of Snf1 and Hxk2. (A) Snf1 abundance is unaffected by varying proteasome capacity. Snf1 protein levels in 40 μg normalized lysates were investigated via a C-terminal FLAG tag in cells with increased (*ubr2Δ*) or decreased (*rpn4Δ*) proteasome activity under fermentative (log) or oxidative growth conditions (PDS). (B) The abundance of Hxk2, a co-repressor for Mig1, is not affected. Hxk2 protein levels in normalized lysates (10 μg) were investigated via a C-terminal FLAG tag in cells with increased (*ubr2Δ*) or decreased (*rpn4Δ, rpn4Δ ubr2Δ*) proteasome activity under fermentative (log) or oxidative growth conditions (PDS). (C) Hxk2 abundance was tested in normalized lysates from WT or mig1*Δ cells*. Detection of PGK1 was used as a loading control. The chemiluminescence signals of the immunoblot analysis were recorded in an Image Quant detection instrument (upper panels) and quantified using the Image Quant software (lower panels). The mean +- SD of three independent experiments is presented.
(TIF)

S7 Fig Mig1 is degraded by the proteasome. The turnover of Mig1 in cells grown in galactose was determined in WT and *ubr2Δ* cells after new synthesis was blocked with 200 μg/ml CHX in the absence or presence of the proteasome-specific inhibitor MG132. Mig1 levels were detected and quantified as described in Fig. 6.
(TIF)

S8 Fig Mitochondrial fragmentation in cells lacking *MIG1* and *MIG2*. Loss of *MIG1* induces mitochondrial hyperfragmentation. To visualize mitochondria, GFP was tagged with a mitochondrial import signal. The vector was introduced in cells deleted for *MIG1*, *MIG2* or *HXK2*. Mitochondrial morphology was recorded via live cell fluorescence microscopy under logarithmic growth conditions. Projected sequential Z-stacks fluorescence images are presented. DIC: differential interference contrast. ~ 240 cells of each strain were analyzed by visual inspection (left panel).
(TIF)

S9 Fig Loss of *MIG1* results in a growth defect in cells overexpressing *SIR2*. Phenotypic analysis of *SIR2* overexpression strains in the absence of *MIG1* or *MIG2*. 5-fold serial dilutions of the strains indicated were spotted on YPD, YPD supplemented with cadmium chloride (upper panels) or on complete media with acetate or glycerol as the sole carbon source (lower panels) and incubated at 30°C.
(TIF)
