# Supplementary material for: Proteasomes, Sir2, and Hxk2 Form an Interconnected Aging Network That Impinges on the AMPK/Snf1-Regulated Transcriptional Repressor Mig1
Source: PLoS Genet. 2015 Jan 28;11(1):e1004968. doi: 10.1371/journal.pgen.1004968 (PMC4309596; doi:10.1371/journal.pgen.1004968)
Supplement: S1 Table — (DOCX) [file pgen.1004968.s001.docx]

**Table S1: Strains used in this study**

| **Strain Name** | ***Genotype*** | **Figure** | **Reference** |
| --- | --- | --- | --- |
| BY4742 | *MATα his3Δ1 leu2Δ0 lys2∆ ura3Δ0* | Fig. 1-6, 9  S2, 4, 5, 9 | [75] |
| KK229 | *MATα his3Δ1 leu2Δ0 lys2∆ ura3Δ0 SIR2-OE(LEU2)* | Fig. 9, S9 | [56] |
| yMS1353 | *MATα his3Δ1 leu2Δ0 met15Δ0 ura3Δ0 MSN2-GFP(HIS3)* | Fig. S3 | This study |
| yMS1355 | *MATα his3Δ1 leu2Δ0 lys2∆ ura3Δ0 MSN2-GFP(HIS3) rpn4∆HphMX* | Fig. S3 | This study |
| yMS1360 | *MATα his3Δ1 leu2Δ0 lys2∆ ura3Δ0 MSN2-GFP(HIS3)* *ubr2ΔNatMX* | Fig. S3 | This study |
| yMS1632 | *MATα his3Δ1 leu2Δ0 lys2∆ ura3Δ0 mig1Δ:: KanMX* | Fig. 4C, 9, S5 | This study |
| yMS1639 | *MATα his3Δ1 leu2Δ0 lys2∆ ura3Δ0 snf4Δ:: KanMX* | Fig. 4B | This study |
| yMS1641 | *MATα his3Δ1 leu2Δ0 lys2∆ ura3Δ0 snf4Δ:: KanMX ubr2ΔNatMX* | Fig. 4B | This study |
| yMS1642 | *MATα his3Δ1 leu2Δ0 lys2∆ ura3Δ0 mig1Δ:: KanMX ubr2ΔNatMX* | Fig. 4C | This study |
| yMS1646 | *MATα his3Δ1 leu2Δ0 lys2∆ ura3Δ0 snf4Δ:: KanMX rpn4∆HphMX* | Fig. 4B | This study |
| yMS1648 | *MATα his3Δ1 leu2Δ0 lys2∆ ura3Δ0 mig1Δ:: KanMX rpn4∆HphMX* | Fig. 4C | This study |
| yMS1652 | *MATα his3Δ1 leu2Δ0 lys2∆ ura3Δ0 hxk2Δ:: KanMX rpn4Δ:: HphMX* | Fig. 9C | This study |
| yMS1654 | *MATα his3Δ1 leu2Δ0 lys2∆ ura3Δ0 hxk2Δ:: KanMX ubr2Δ:: HphMX* | Fig. 9C | This study |
| yMS1689 | *MAT***a** *his3Δ1 leu2Δ0 ura3Δ0*  *SNF1-FLAG (HphMX)* | Fig. S6A | This study |
| yMS1693 | *MAT****a*** *his3Δ1 leu2Δ0 met15Δ0 ura3Δ0 SNF1-FLAG (HphMX) rpn4Δ:: HphMX* | Fig. S4A | This study |
| yMS1695 | *MAT****a*** *his3Δ1 leu2Δ0 lys2∆ ura3Δ0 SNF1-FLAG (HphMX) ubr2Δ::NatMX* | Fig. S4A | This study |
| yMS1711 | *MATα his3Δ1 leu2Δ0 lys2∆ met15Δ0 ura3Δ0 rpn4∆HphMX ubr2∆NatMX* | Fig. 1-3, S2 | This study |
| yMS1773 | *MATα his3Δ1 leu2Δ0 lys2∆ ura3Δ0 ubr2∆NatMX* | Fig. 1-5, 9, S2, 4, 7 | This study |
| yMS1776 | *MATα his3Δ1 leu2Δ0 lys2∆ ura3Δ0 rpn4∆HphMX* | Fig. 1-5, 9, S2, 4 | This study |
| yMS1797 | *MATα his3Δ1 leu2Δ0 lys2∆ ura3Δ0 pCox4-GFP(HIS3)* | Fig. 2A, S1, 8 | This study |
| yMS1800 | *MATα his3Δ1 leu2Δ0 lys2∆ ura3Δ0 ubr2∆NatMX pCox4-GFP(HIS3)* | Fig. 2A, S1 | This study |
| yMS1801 | *MATα his3Δ1 leu2Δ0 lys2∆ ura3Δ0 rpn4∆HphMX pCox4-GFP(HIS3)* | Fig. 2A,S1 | This study |
| yMS1847 | *MATα his3Δ1 leu2Δ0 lys2∆ met15Δ0 ura3Δ0 fzo1∆::KanMX*  *pCox4-GFP(HIS3)* | Fig. 2A,S1 | This study |
| yMS1915 | *MATα his3Δ1 leu2Δ0 lys2∆ ura3Δ0 snf1∆KanMX* | Fig. 4A, S4B | This study |
| yMS1917 | *MATα his3Δ1 leu2Δ0 lys2∆ ura3Δ0 ubr2∆NatMX snf1∆KanMX* | Fig. 4A, S4B | This study |
| yMS1919 | *MATα his3Δ1 leu2Δ0 lys2∆ ura3Δ0 rpn4∆HphMX snf1∆KanMX* | Fig. 4A, S4B | This study |
| yMS1930 | *MATα his3Δ1 leu2Δ0 ura3Δ0 mig2∆KanMX* | Fig. 4D | This study |
| yMS1932 | *MATα his3Δ1 leu2Δ0 lys2∆ ura3Δ0 mig2∆KanMX rpn4∆HphMX* | Fig. 4D | This study |
| yMS1934 | *MATα his3Δ1 leu2Δ0 ura3Δ0 mig2∆KanMX ubr2∆NatMX* | Fig. 4D | This study |
| yMS1984 | *MATα his3Δ1 leu2Δ0 ura3Δ0 MIG1-GFP(HIS3) ubr2∆NatMX* | Fig. 7 | This study |
| yMS1986 | *MATα his3Δ1 leu2Δ0 met15Δ0 lys2∆0 ura3Δ0 MIG1-GFP(HIS3) rpn4∆HphMX* | Fig. 7 | This study |
| yMS1988 | *MATα his3Δ1 leu2Δ0 met15Δ0 ura3Δ0 MIG1-GFP(HIS3) rpn4∆HphMX ubr2∆NatMX* | Fig. 7 | This study |
| yMS1990 | *MATα his3Δ1 leu2Δ0 met15Δ0 ura3Δ0 MIG1-GFP(HIS3)* | Fig. 7 | This study |
| yMS2044 | *MAT****a*** *his3Δ1 leu2Δ0 met15Δ0 ura3Δ0 HXK2-HA3(HIS3)* | Fig. S4B | This study |
| yMS2046 | *MAT****a*** *his3Δ1 leu2Δ0 ura3Δ0 HXK2-HA3(HIS3) rpn4Δ::HphMX* | Fig. S4B | This study |
| yMS2048 | *MAT****a*** *his3Δ1 leu2Δ0 ura3Δ0 HXK2-HA3(HIS3) ubr2Δ::NatMX* | Fig. S4B | This study |
| yMS2050 | *MAT****a*** *his3Δ1 leu2Δ0 lys2Δ0 ura3Δ0 HXK2-HA3(HIS3) rpn4Δ::HphMX ubr2Δ::NatMX* | Fig. S4B | This study |
| yMS2106 | *MAT***α** *his3Δ1 leu2Δ0 lys2∆ ura3Δ0 MSN2-GFP(HIS3) rpn4∆HphMX ubr2∆NatMX* | Fig. S3 | This study |
| yMS2110 | *MAT****a*** *his3Δ1 leu2Δ0 met15Δ0 ura3Δ0 MIG1-HA3(KanMX)* | Fig. 5, S6 | This study |
| yMS2111 | *MAT****a*** *his3Δ1 leu2Δ0 met15Δ0 ura3Δ0 MIG1-HA3(KanMX) ubr2Δ::NatMX* | Fig. 5, S6 | This study |
| yMS2112 | *MAT****a*** *his3Δ1 leu2Δ0 met15Δ0 ura3Δ0 MIG1-HA3(KanMX) rpn4Δ::HphMX* | Fig. 5 | This study |
| yMS2113 | *MAT****a*** *his3Δ1 leu2Δ0 met15Δ0 ura3Δ0 MIG1-HA3(KanMX) rpn4Δ::HphMX ubr2Δ::NatMX* | Fig. 5 | This study |
| yMS2219 | *MAT****a*** *his3Δ1 leu2Δ0 met15Δ0 ura3Δ0 FZO1-HA3(HIS3)* | Fig. S2A | This study |
| yMS2220 | *MAT****a*** *his3Δ1 leu2Δ0 met15Δ0 ura3Δ0 FZO1-HA3(HIS3) ubr2Δ::NatMX* | Fig. S2A | This study |
| yMS2221 | *MAT****a*** *his3Δ1 leu2Δ0 met15Δ0 ura3Δ0 FZO1-HA3(HIS3) rpn4Δ::HphMX* | Fig. S2A | This study |
| yMS2229 | *MATα his3Δ1 leu2Δ0 ura3Δ0 MIG1-GFP(HIS3) mito-dsRed (URA3)* | Fig. 8 | This study |
| yMS2230 | *MATα his3Δ1 leu2Δ0 met15Δ0 lys2∆0 ura3Δ0 MIG1-GFP(HIS3) rpn4∆HphMX*  *mito-dsRed (URA3)* | Fig. 8 | This study |
| yMS2231 | *MATα his3Δ1 leu2Δ0 lys2∆0 ura3Δ0 MIG1-GFP(HIS3) ubr2∆NatMX*  *mito-dsRed (URA3)* | Fig. 8 | This study |
| yMS2250 | *MATα his3Δ1 leu2Δ0 ura3Δ0 mig1∆KanMX pCox4-GFP(HIS3)* | Fig. S8 | This study |
| yMS2251 | *MATα his3Δ1 leu2Δ0 lys2∆0 ura3Δ0 hxk2∆KanMX pCox4-GFP(HIS3)* | Fig. S8 | This study |
| yMS2252 | *MATα his3Δ1 leu2Δ0 lys2∆0 ura3Δ0 mig2∆KanMX pCox4-GFP(HIS3)* | Fig. S8 | This study |
| yMS2257 | *MAT*α *his3Δ1 leu2Δ0 lys2∆ ura3Δ0*  *mig2∆::NatMX* | Fig. S5, 9 | This study |
| yMS2258 | *MAT*α *his3Δ1 leu2Δ0 lys2∆ ura3Δ0*  *mig1∆::KanMX mig2∆::NatMX* | Fig. S5 | This study |
| yMS2260 | *MAT*α *his3Δ1 leu2Δ0 lys2∆ ura3Δ0*  *hxk2∆::NatMX* | Fig. 9 | This study |
| yMS2261 | *MAT*α *his3Δ1 leu2Δ0 lys2∆ ura3Δ0*  *mig1∆::KanMX hxk2∆::NatMX* | Fig. 9A | This study |
| yMS2266 | *MAT*α *his3Δ1 leu2Δ0 lys2∆ ura3Δ0*  *SIR2-OE(LEU2) mig2∆::NatMX* | Fig. S8 | This study |
| yMS2267 | *MAT*α *his3Δ1 leu2Δ0 lys2∆ ura3Δ0*  *SIR2-OE(LEU2) mig1∆::NatMX* | Fig. 9B, S9 | This study |
| yMS2268 | *MAT*α *his3Δ1 leu2Δ0 lys2∆ ura3Δ0*  *mig1∆::NatMX* | Fig. 9B, S9 | This study |
| yMS2269 | *MAT*α *his3Δ1 leu2Δ0 lys2∆ ura3Δ0*  *SIR2-OE(LEU2) sip2∆::NatMX* | Fig. 9B | This study |
| yMS2270 | *MAT*α *his3Δ1 leu2Δ0 lys2∆ ura3Δ0*  *sip2∆::NatMX* | Fig. 9B | This study |
| yMS2277 | *MATα his3Δ1 leu2Δ0 ura3Δ0 MIG1-GFP(HIS3) hxk2∆NatMX*  *mito-dsRed (URA3)* | Fig. 8 | This study |
| yMS2324 | *MATα his3Δ1 leu2Δ0 lys2∆0 ura3Δ0 MIG1-GFP(HIS3) SIR2-OE(LEU2)*  *mito-dsRed (URA3)* | Fig. 8 | This study |
| yMS2361 | *MATα his3Δ1 leu2Δ0 lys2∆ ura3Δ0*  *mig3∆HphMX* | Fig. S5 | This study |
| yMS2362 | *MATα his3Δ1 leu2Δ0 lys2∆ ura3Δ0*  *mig3∆HphMX mig1∆::KanMX mig2∆::NatMX* | Fig. S5 | This study |
| yMS2365 | *MATα his3Δ1 leu2Δ0 lys2∆ ura3Δ0*  *TEF1pMIG1::KanMX* | Fig. 6A | This study |
| yMS2366 | *MATα his3Δ1 leu2Δ0 lys2∆ ura3Δ0*  *TEF1pMIG1::KanMX rpn4Δ::HphMX* | Fig. 6A | This study |
| yMS2367 | *MATα his3Δ1 leu2Δ0 lys2∆ ura3Δ0*  *TEF1pMIG1::KanMX* *ubr2Δ::NatMX* | Fig. 6A | This study |
| yMS2371 | *MATα his3Δ1 leu2Δ0 lys2∆ ura3Δ0*  *Mig1S311A* | Fig. 6B | This study |
| yMS2372 | *MATα his3Δ1 leu2Δ0 lys2∆ ura3Δ0*  *Mig1S311A rpn4Δ::HphMX* | Fig. 6B | This study |
| yMS2373 | *MATα his3Δ1 leu2Δ0 lys2∆ ura3Δ0*  *Mig1S311A ubr2Δ::NatMX* | Fig. 6B | This study |
| yMS2377 | *MATα his3Δ1 leu2Δ0 lys2∆ ura3Δ0*  *mig3∆HphMX mig1∆::KanMX* | Fig. S5 | This study |
| yMS2382 | *MAT***α** *his3Δ1 leu2Δ0 met15Δ0 ura3Δ0 pdr5Δ::HphMX MIG1-HA (HIS3)* | Fig. S7 | This study |
| yMS2384 | *MAT****a*** *his3Δ1 leu2Δ0 met15Δ0 ura3Δ0 ubr2Δ::NatMX pdr5Δ::HphMX MIG1-HA (HIS3)* | Fig. S7 | This study |
| ST2889 | rho^O^ *MATα his3Δ1 leu2Δ0 met15Δ0 ura3Δ0* | Fig.1C | This study |
| ST2890 | rho^O^ *MATα his3Δ1 leu2Δ0 met15Δ0 ura3Δ0 ubr2∆NatMX* | Fig. 1C | This study |
| ST2891 | rho^O^ *MATα his3Δ1 leu2Δ0 met15Δ0 ura3Δ0 rpn4∆HphMX* | Fig. 1C | This study |
| ST2892 | rho^O^ *MATα his3Δ1 leu2Δ0 met15Δ0 ura3Δ rpn4∆HphMX ubr2∆NatMX* | Fig. 1C | This study |
| ST3131 | *MAT*α *his3Δ1 leu2Δ0 lys2∆ ura3Δ0 sip2∆::KanMX ubr2∆::NatMX* | Fig. S4A | This study |
| ST3138 | *MAT*α *his3Δ1 leu2Δ0 lys2∆ ura3Δ0 gal83∆::KanMX ubr2∆::NatMX* | Fig. S4A | This study |
| DC122F1 | *MAT*α *his3Δ1 leu2Δ0 lys2∆ ura3Δ0 sip2∆::KanMX* | Fig. S3A | This study |
| DC144C4 | *MAT*α *his3Δ1 leu2Δ0 lys2∆ ura3Δ0 gal83∆::KanMX* | Fig. S4A | This study |
| BR2227 | *MATα his3Δ1 leu2Δ0 met15Δ0 ura3Δ0 SIR2-OE(LEU2) rpn4∆HphMX* | Fig. 9D | This study |
| BR2280 | *MATα his3Δ1 leu2Δ0 met15Δ0 ura3Δ0*  *SIR2-OE(LEU2) ubr2∆NatMX* | Fig. 9D | This study |
