# Supplementary material for: Proteasomes, Sir2, and Hxk2 Form an Interconnected Aging Network That Impinges on the AMPK/Snf1-Regulated Transcriptional Repressor Mig1
Source: PLoS Genet. 2015 Jan 28;11(1):e1004968. doi: 10.1371/journal.pgen.1004968 (PMC4309596; doi:10.1371/journal.pgen.1004968)
Supplement: S2 Table — (DOCX) [file pgen.1004968.s002.docx]

**Table S2: Primers used for qRT-PCR**

| Primer name | Sequence |
| --- | --- |
| ACT1-F | GTTGCTGCTTTGGTTATTGA |
| ACT1-R | CATACCGACCATGATACCTT |
| SUC2-F | CACCACACAAACCATATCCAA |
| SUC2-R | TCTAAACCCTTGAACCAAAGTGA |
| HXK1-F | TCCTTCGATAATGAACATTTGGT |
| HXK1-R | TGTTCGTCGACAGCAACATC |
| GAL1-F | ACAATGGCGGTATGGATCA |
| GAL1-R | GCGGTTTGAACTCAACGTATAGA |
