# Supplementary material for: Proteasomes, Sir2, and Hxk2 Form an Interconnected Aging Network That Impinges on the AMPK/Snf1-Regulated Transcriptional Repressor Mig1
Source: PLoS Genet. 2015 Jan 28;11(1):e1004968. doi: 10.1371/journal.pgen.1004968 (PMC4309596; doi:10.1371/journal.pgen.1004968)
Supplement: S3 Table — P-value matrices for Figs. 1, 4, 6, and 9 and S4A and S5 Figs. P-values were assessed by a Wilcoxon test. Ranksum p-values: * p< 0.05, ** p < 0.01, *** p < 0.001, ns = not significant L: Left panel; R: Right panel. (DOCX) [file pgen.1004968.s003.docx]

**Table S3: Statistical analysis of the RLS experiments presented.**

| **Figure 1A** | **WT** | ***ubr2∆*** | ***rpn4∆*** |
| --- | --- | --- | --- |
| ***ubr2∆*** | ******* |  |  |
| ***rpn4∆*** | ******* | ******* |  |
| ***ubr2∆ rpn4∆*** | ******* | ******* | **ns** |

| **Figure 1B (L)** | **WT** | ***ubr2∆*** | ***rpn4∆*** |
| --- | --- | --- | --- |
| ***ubr2∆*** | ******* |  |  |
| ***rpn4∆*** | ******* | ******* |  |
| ***ubr2∆ rpn4∆*** | ******* | ******* | ****** |

| **Figure 1B (R)** | **WT** | ***ubr2∆*** | ***rpn4∆*** |
| --- | --- | --- | --- |
| ***ubr2∆*** | ******* |  |  |
| ***rpn4∆*** | ******* | ******* |  |
| ***ubr2∆ rpn4∆*** | ****** | ******* | ***** |

| **Figure 1C (L)** | **WT** | ***ubr2∆*** | ***rpn4∆*** |
| --- | --- | --- | --- |
| ***ubr2∆*** | ****** |  |  |
| ***rpn4∆*** | ***** | ******* |  |
| ***ubr2∆ rpn4∆*** | **ns** | ****** | ****** |

| **Figure 1C (R)** | **rho^0^** | ***ubr2∆*** | ***rpn4∆*** |
| --- | --- | --- | --- |
| **rho^0^ *ubr2∆*** | ******* |  |  |
| **rho^0^ *rpn4∆*** | **ns** | ******* |  |
| **rho^0^ *ubr2∆ rpn4∆*** | ****** | **ns** | **ns** |

| **Figure 4A (L)** | **WT** | ***ubr2∆*** | ***snf1∆*** |
| --- | --- | --- | --- |
| ***ubr2∆*** | ******* |  |  |
| ***snf1∆*** | ***** | ******* |  |
| ***snf1∆ ubr2∆*** | ******* | ******* | **ns** |

| **Figure 4A (R)** | **WT** | ***snf1∆*** | ***rpn4∆*** |
| --- | --- | --- | --- |
| ***snf1∆*** | **ns** |  |  |
| ***rpn4∆*** | ******* | ******* |  |
| ***snf1∆ rpn4∆*** | ******* | ******* | ******* |

| **Figure 4B (L)** | **WT** | ***ubr2∆*** | ***snf4∆*** |
| --- | --- | --- | --- |
| ***ubr2∆*** | ******* |  |  |
| ***snf4∆*** | **ns** | ******* |  |
| ***snf4∆ ubr2∆*** | **ns** | ******* | **ns** |

| **Figure 4B (R)** | **WT** | ***snf4∆*** | ***rpn4∆*** |
| --- | --- | --- | --- |
| ***snf4∆*** | ***** |  |  |
| ***rpn4∆*** | ******* | ******* |  |
| ***snf4∆ rpn4∆*** | ******* | ******* | ******* |

| **Figure 4C (L)** | **WT** | ***ubr2∆*** | ***mig1∆*** |
| --- | --- | --- | --- |
| ***ubr2∆*** | ******* |  |  |
| ***mig1∆*** | **ns** | ******* |  |
| ***mig1∆ ubr2∆*** | **ns** | ******* | **ns** |

| **Figure 4C (R)** | **WT** | ***rpn4∆*** | ***mig1∆*** |
| --- | --- | --- | --- |
| ***rpn4∆*** | ******* |  |  |
| ***mig1∆*** | **ns** | ******* | ******* |
| ***mig1∆ rpn4∆*** | ******* | **ns** |  |

| **Figure 4D (L)** | **WT** | ***ubr2∆*** | ***mig2∆*** |
| --- | --- | --- | --- |
| ***ubr2∆*** | ******* |  |  |
| ***mig2∆*** | ***** | ******* |  |
| ***mig2∆ ubr2∆*** | ****** | ******* | **ns** |

| **Figure 4D (R)** | **WT** | ***rpn4∆*** | ***mig2∆*** |
| --- | --- | --- | --- |
| ***rpn4∆*** | ******* |  |  |
| ***mig2∆*** | ***** | ******* |  |
| ***mig2∆ rpn4∆*** | ******* | **ns** | ******* |

| **Figure 6A (L)** | **WT** | ***ubr2∆*** | ***TEFpMIG1*** |
| --- | --- | --- | --- |
| ***ubr2∆*** | ******* |  |  |
| ***TEFpMIG1*** | ******* | ******* |  |
| ***ubr2∆ TEFpMIG1*** | ******* | ******* | **ns** |

| **Figure 6A (R)** | **WT** | ***rpn4∆*** | ***TEFpMIG1*** |
| --- | --- | --- | --- |
| ***rpn4∆*** | ******* |  |  |
| ***TEFpMIG*** | ******* | ******* |  |
| ***rpn4∆ TEFpMIG1*** | ******* | **ns** | ******* |

| **Figure 6B (L)** | **WT** | ***ubr2∆*** | ***mig1S311A*** |
| --- | --- | --- | --- |
| ***ubr2∆*** | ******* |  |  |
| ***mig1S311A*** | ******* | ******* |  |
| ***ubr2∆ mig1S311A*** | ******* | ******* | ******* |

| **Figure 6B (R)** | **WT** | ***rpn4∆*** | ***mig1S311A*** |
| --- | --- | --- | --- |
| ***rpn4∆*** | ******* |  |  |
| ***mig1S311A*** | *********** | ****** |  |
| ***rpn4∆ mig1S311A*** | ******* | ****** | **ns** |

| **Figure 9A** | **WT** | ***hxk2∆*** | ***mig1∆*** |
| --- | --- | --- | --- |
| ***hxk2∆*** | ***** |  |  |
| ***mig1∆*** | **ns** | ***** |  |
| ***hxk2∆ mig1∆*** | ****** | **ns** | ****** |

| **Figure 9B (L)** | **WT** | ***SIR2-OE*** | ***mig1∆*** |
| --- | --- | --- | --- |
| ***SIR2-OE*** | ******* |  |  |
| ***mig1∆*** | **ns** | ******* |  |
| ***SIR2-OE mig1∆*** | ****** | **ns** | **ns** |

| **Figure 9B (R)** | **WT** | ***SIR2-OE*** | ***sip2∆*** |
| --- | --- | --- | --- |
| ***SIR2-OE∆*** | ******* |  |  |
| ***sip2∆*** | **ns** | ******* |  |
| ***SIR2-OE sip2∆*** | **ns** | ****** | **ns** |

| **Figure 9C (L)** | **WT** | ***ubr2∆*** | ***hxk2∆*** |
| --- | --- | --- | --- |
| ***ubr2∆*** | ****** |  |  |
| ***hxk2∆*** | **ns** | **ns** |  |
| ***hxk2∆ ubr2∆*** | ******* | ***** | ****** |

| **Figure 9C (R)** | **WT** | ***rpn4∆*** | ***hxk2∆*** |
| --- | --- | --- | --- |
| ***rpn4∆*** | ******* |  |  |
| ***hxk2∆*** | **ns** | ******* |  |
| ***hxk2∆ rpn4∆*** | ****** | **ns** | ******* |

| **Figure 9D (L)** | **WT** | ***SIR2-OE*** | ***ubr2∆*** |
| --- | --- | --- | --- |
| ***SIR2-OE*** | ******* |  |  |
| ***ubr2∆*** | ****** | **ns** |  |
| ***SIR2-OE ubr2∆*** | ******* | **ns** | ***** |

| **Figure 9D (R)** | **WT** | ***SIR2-OE*** | ***rpn4∆*** |
| --- | --- | --- | --- |
| ***SIR2-OE*** | ******* |  |  |
| ***rpn4∆*** | ******* | ******* |  |
| ***SIR2-OE rpn4∆*** | ******* | ******* | **ns** |

| **Suppl. Figure 4A (L)** | **WT** | ***ubr2∆*** | ***sip2∆*** |
| --- | --- | --- | --- |
| ***ubr2∆*** | ******* |  |  |
| ***sip2∆*** | **ns** | ******* |  |
| ***ubr2∆ sip2∆*** | ******* | **ns** | ******* |

| **Suppl. Figure 4A (R)** | **WT** | ***ubr2∆*** | ***gal83∆*** |
| --- | --- | --- | --- |
| ***ubr2∆*** | ******* |  |  |
| ***gal83∆*** | ******* | ******* |  |
| ***ubr2∆ gal83∆*** | ******* | **ns** | ******* |

| **Suppl. Figure 5A** | **WT** | ***mig1∆*** | ***mig2∆*** |
| --- | --- | --- | --- |
| ***mig1∆*** | **ns** |  |  |
| ***mig2∆*** | **ns** | **ns** |  |
| ***mig2∆ mig1∆*** | **ns** | **ns** | ****** |

| **Suppl. Figure 5B** | **WT** | ***mig1∆*** | ***mig3∆*** |
| --- | --- | --- | --- |
| ***mig1∆*** | **ns** |  |  |
| ***mig3∆*** | ******* | ******* |  |
| ***mig3∆ mig1∆*** | ******* | *********** | **ns** |

| **Suppl. Figure 5C** | **WT** | ***mig1∆*** | ***mig2∆*** | ***mig3∆*** |
| --- | --- | --- | --- | --- |
| ***mig1∆*** | **ns** |  |  |  |
| ***mig2∆*** | **ns** | **ns** |  |  |
| ***mig3∆*** | ******* | ******* | ******* |  |
| ***mig2∆ mig1∆ mig3∆*** | ******* | ******* | ******* | **ns** |
