# Supplementary figures and images for: Proteasomes, Sir2, and Hxk2 Form an Interconnected Aging Network That Impinges on the AMPK/Snf1-Regulated Transcriptional Repressor Mig1
Source: PLoS Genet. 2015 Jan 28;11(1):e1004968. doi: 10.1371/journal.pgen.1004968 (PMC4309596; doi:10.1371/journal.pgen.1004968)

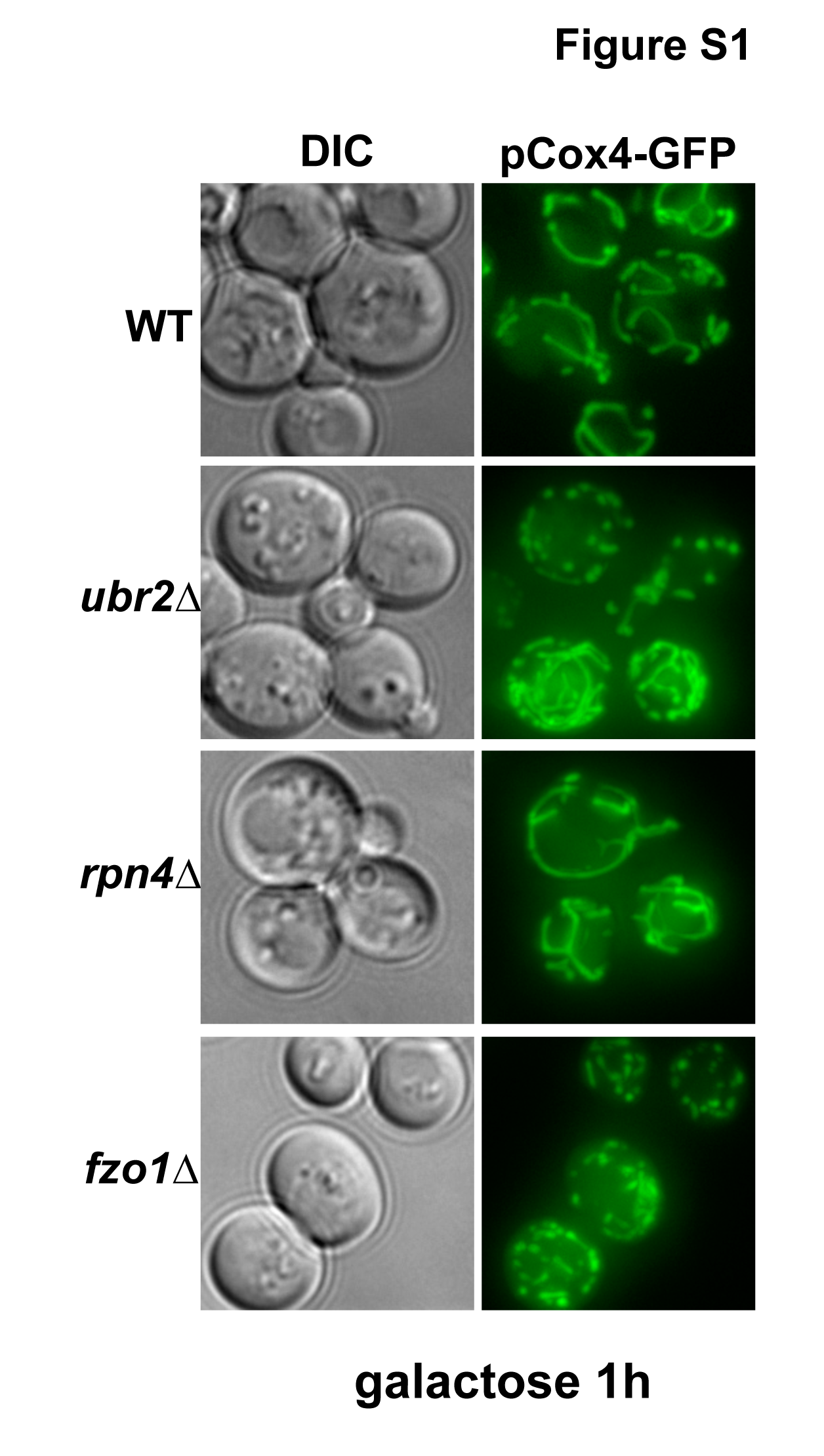

Supplement: S1 Fig — To visualize mitochondria GFP was tagged with a mitochondrial import signal and introduced in cells with increased (ubr2Δ), decreased (rpn4Δ) proteasome capacity and in cells deleted for FZO1. Projected sequential Z-stacks fluorescence images are presented. DIC: differential interference contrast. (TIF) [file pgen.1004968.s004.tif]

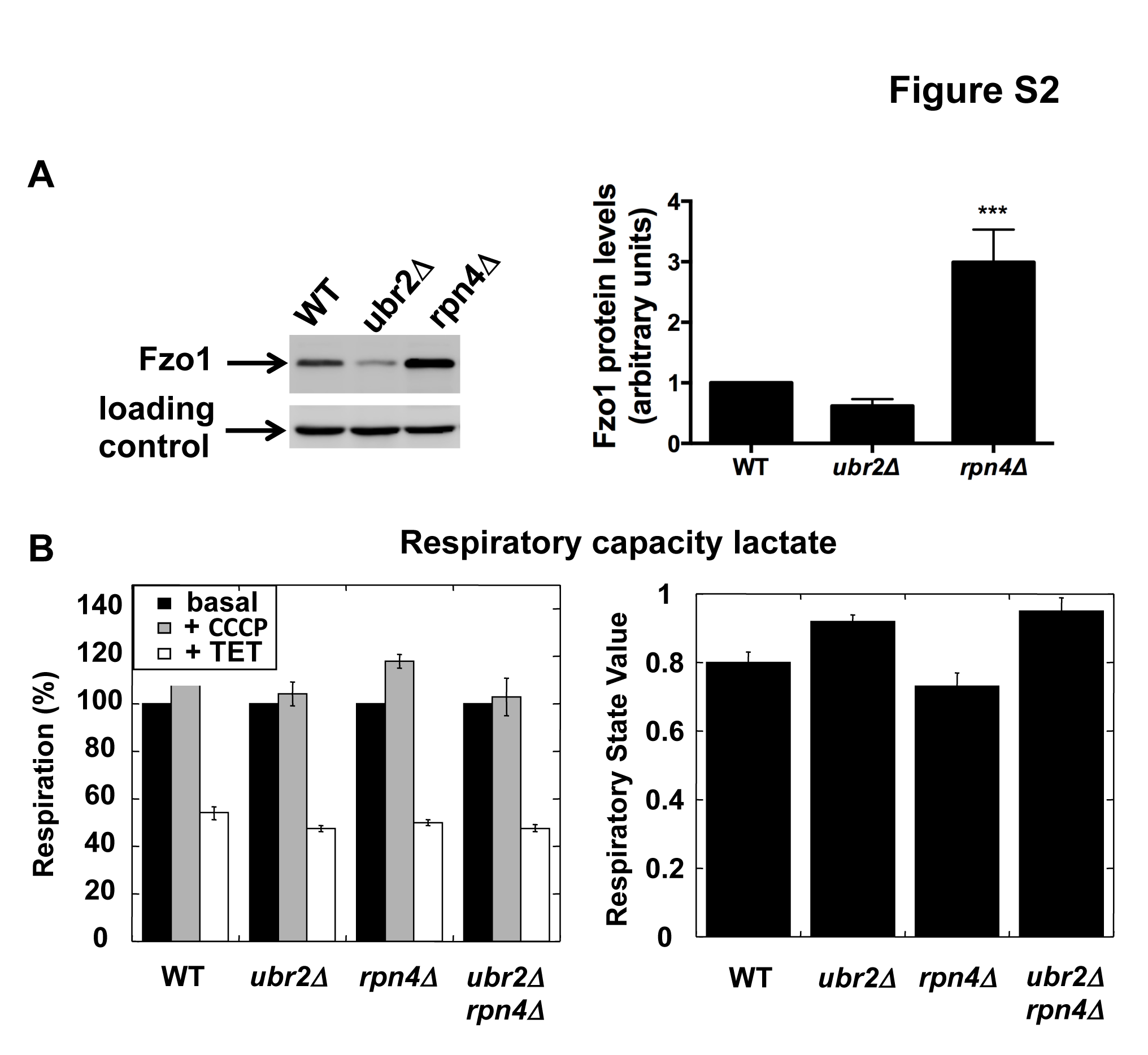

Supplement: S2 Fig — (A) The levels of the mitochondrial fusion protein Fzo1 correlate with proteasome abundance. Fzo1 protein levels were investigated in 20 μg normalized lysates from the strains indicated via a C-terminal HA tag utilizing HA specific antibodies. Detection of PGK1 was used as a loading control. The chemiluminescence signals of the immunoblot analysis were recorded in an Image Quant detection instrument (upper panels) and quantified using the Image Quant software (lower panels). The mean +- SD of three independent experiments is presented. P-values represent the statistical significance relative to WT expression and were assessed by an Ordinary one-way Annova using the GraphPad Prism software. P-values: * p < 0.05, ** p < 0.01, *** p < 0.001. (B) Mitochondrial activity is not affected by proteasome abundance in media containing the non-fermentable carbon source lactate. Experimental procedure as described in Fig. 2A. (TIF) [file pgen.1004968.s005.tif]

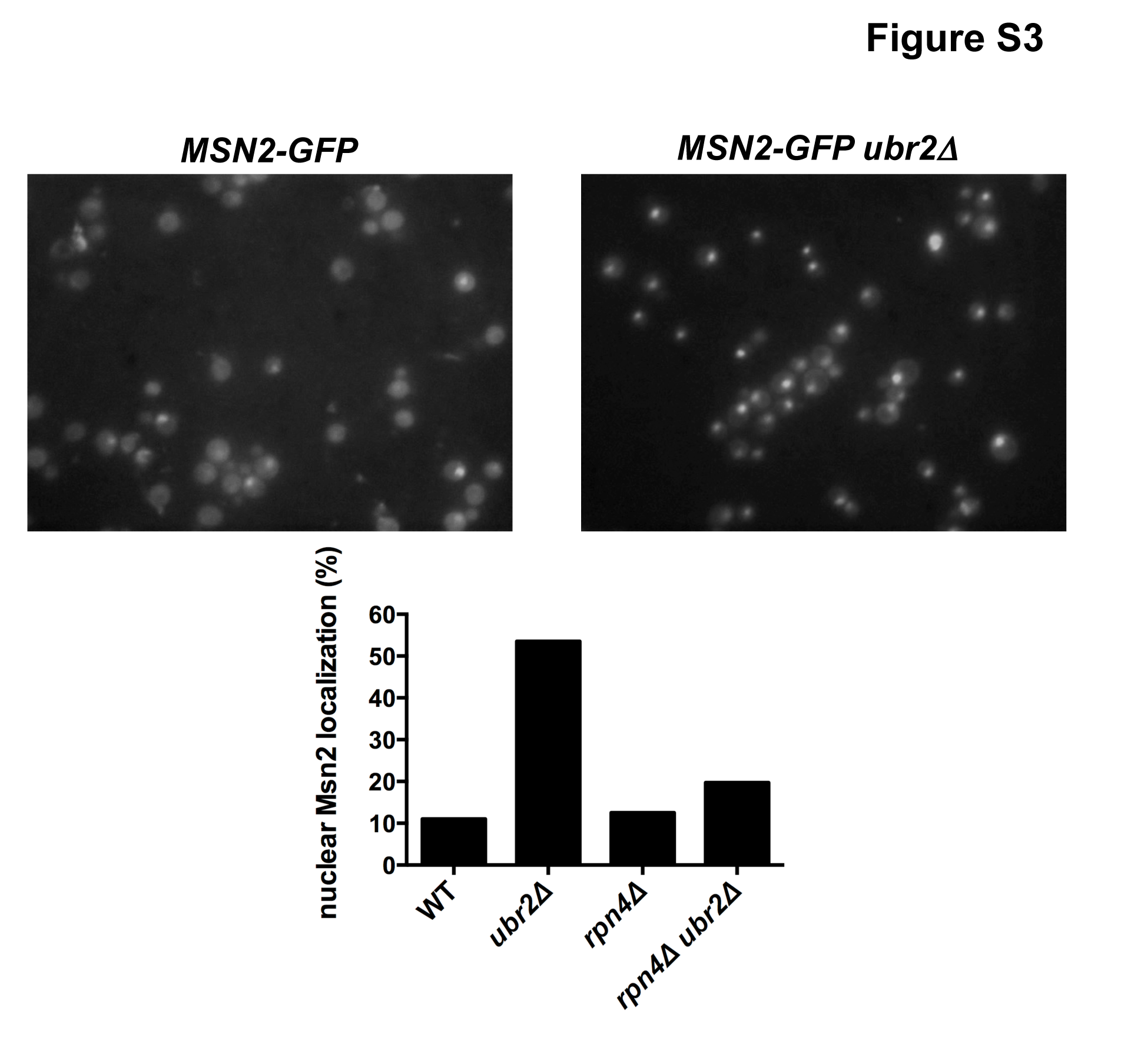

Supplement: S3 Fig — Upper panels: The localization of Msn2 in WT or ubr2Δ cells was visualized via a C-terminal GFP tag with live cell fluorescence microscopy. Lower panel: Quantification of cells with nuclear Msn2 localization was performed via visual inspection of ~ 300 cells in WT, rpn4Δ, ubr2Δ, or rpn4Δ ubr2Δ cells. (TIF) [file pgen.1004968.s006.tif]

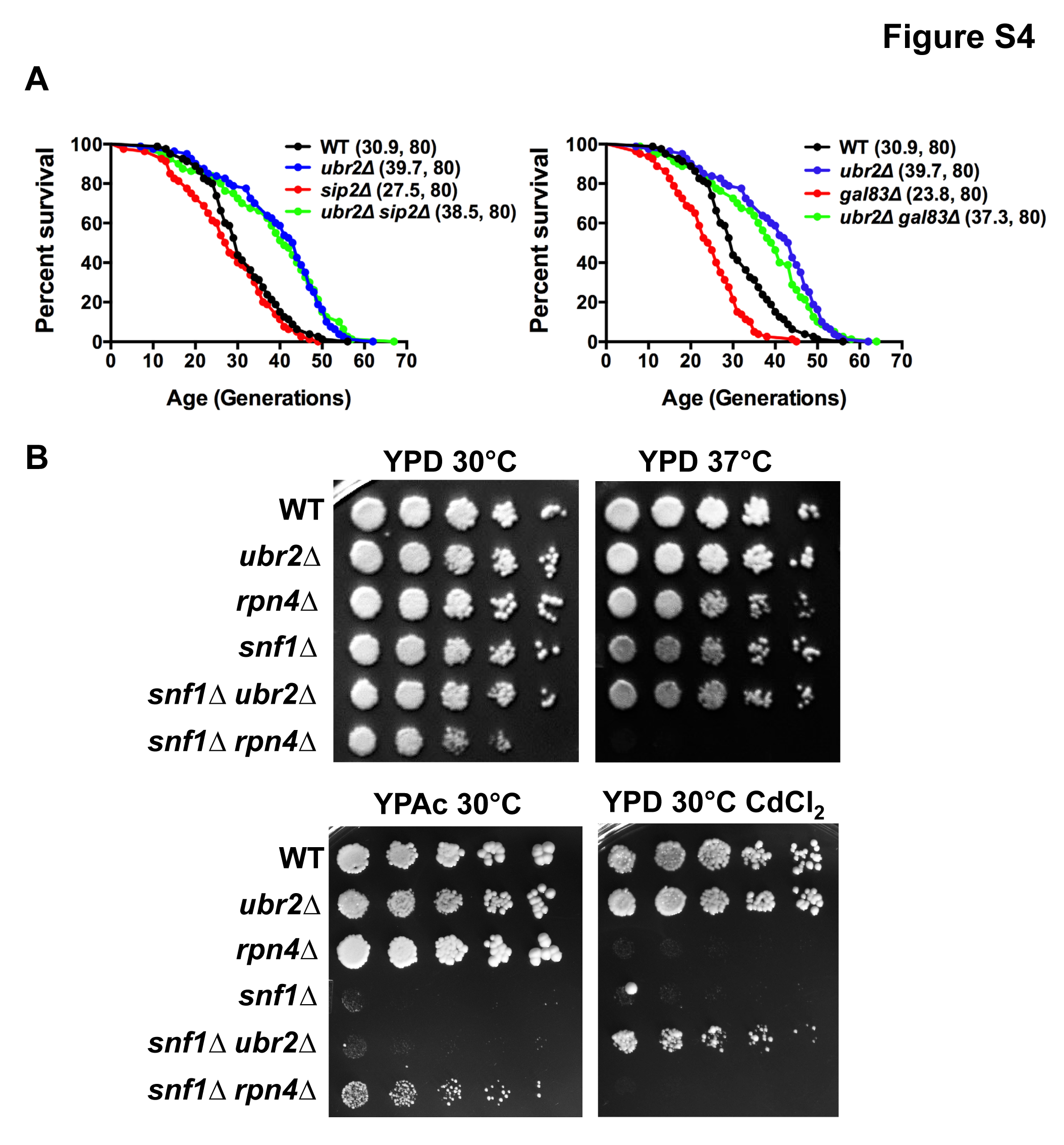

Supplement: S4 Fig — (A) Cells with increased proteasome abundance rescue the short lifespan of cells deleted for the Snf1 β-subunits GAL83 and SIP2. Survival curves of WT cells were compared to ubr2Δ, gal83Δ, and ubr2Δ gal83Δ. Right panel: Survival curves of WT cells were compared to ubr2Δ, sip2Δ, and ubr2Δ sip2Δ. Mean lifespan and cell counts are shown in parenthesis. A statistical analysis of the data is summarized in S3 Table. (B) Phenotypic analysis of proteasome mutants deleted for SNF1: 5-fold serial dilutions of the strains indicated were spotted on YPD and incubated at 30°C or 37°C (upper panels or were spotted on media with acetate as the sole carbon source or on YPD supplemented with cadmium chloride. (TIF) [file pgen.1004968.s007.tif]

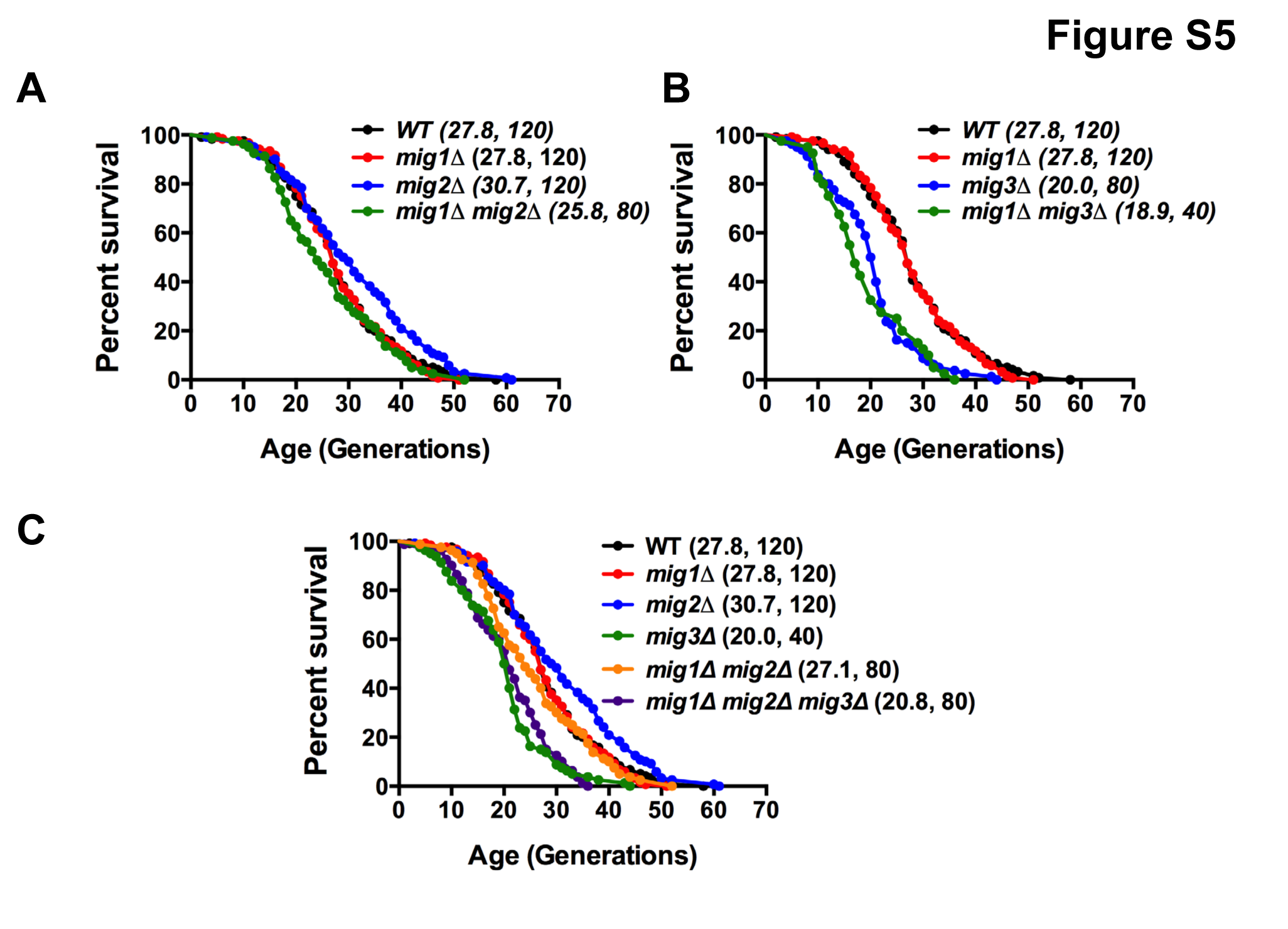

Supplement: S5 Fig — Survival curves of WT cells were compared to (A) mig1Δ, mig2Δ, and mig1Δ mig2Δ; (B) mig1Δ, mig3Δ, and mig1Δ mig3Δ and (C) mig1Δ, mig2Δ, mig3Δ, mig1Δ mig2Δ and mig1Δ mig2Δ mig3Δ. Mean lifespan and cell counts are shown in parenthesis. A statistical analysis of the data is summarized in S3 Table. (TIF) [file pgen.1004968.s008.tif]

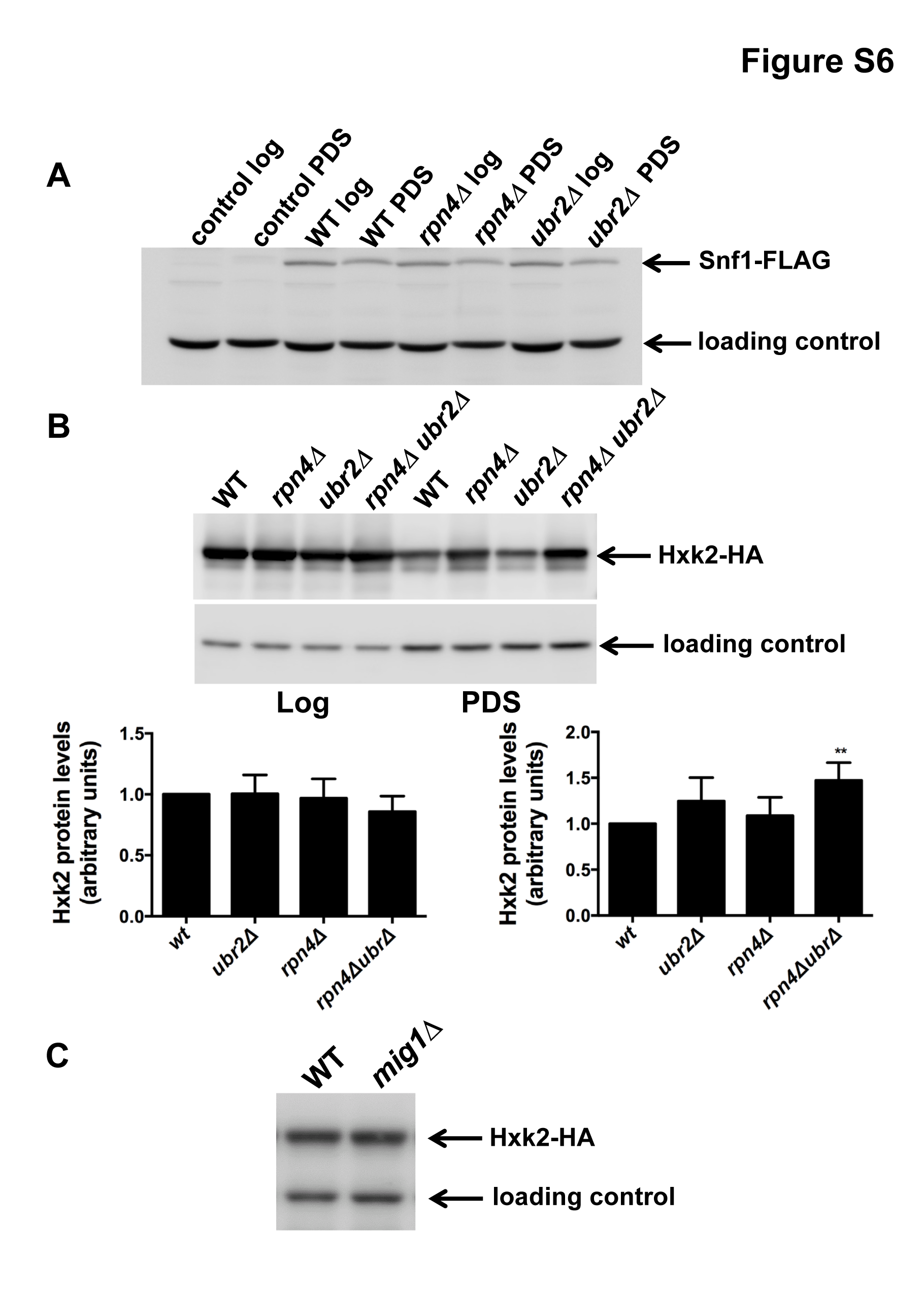

Supplement: S6 Fig — (A) Snf1 abundance is unaffected by varying proteasome capacity. Snf1 protein levels in 40 μg normalized lysates were investigated via a C-terminal FLAG tag in cells with increased (ubr2Δ) or decreased (rpn4Δ) proteasome activity under fermentative (log) or oxidative growth conditions (PDS). (B) The abundance of Hxk2, a co-repressor for Mig1, is not affected. Hxk2 protein levels in normalized lysates (10 μg) were investigated via a C-terminal FLAG tag in cells with increased (ubr2Δ) or decreased (rpn4Δ, rpn4Δ ubr2Δ) proteasome activity under fermentative (log) or oxidative growth conditions (PDS). (C) Hxk2 abundance was tested in normalized lysates from WT or mig1Δ cells. Detection of PGK1 was used as a loading control. The chemiluminescence signals of the immunoblot analysis were recorded in an Image Quant detection instrument (upper panels) and quantified using the Image Quant software (lower panels). The mean +- SD of three independent experiments is presented. (TIF) [file pgen.1004968.s009.tif]

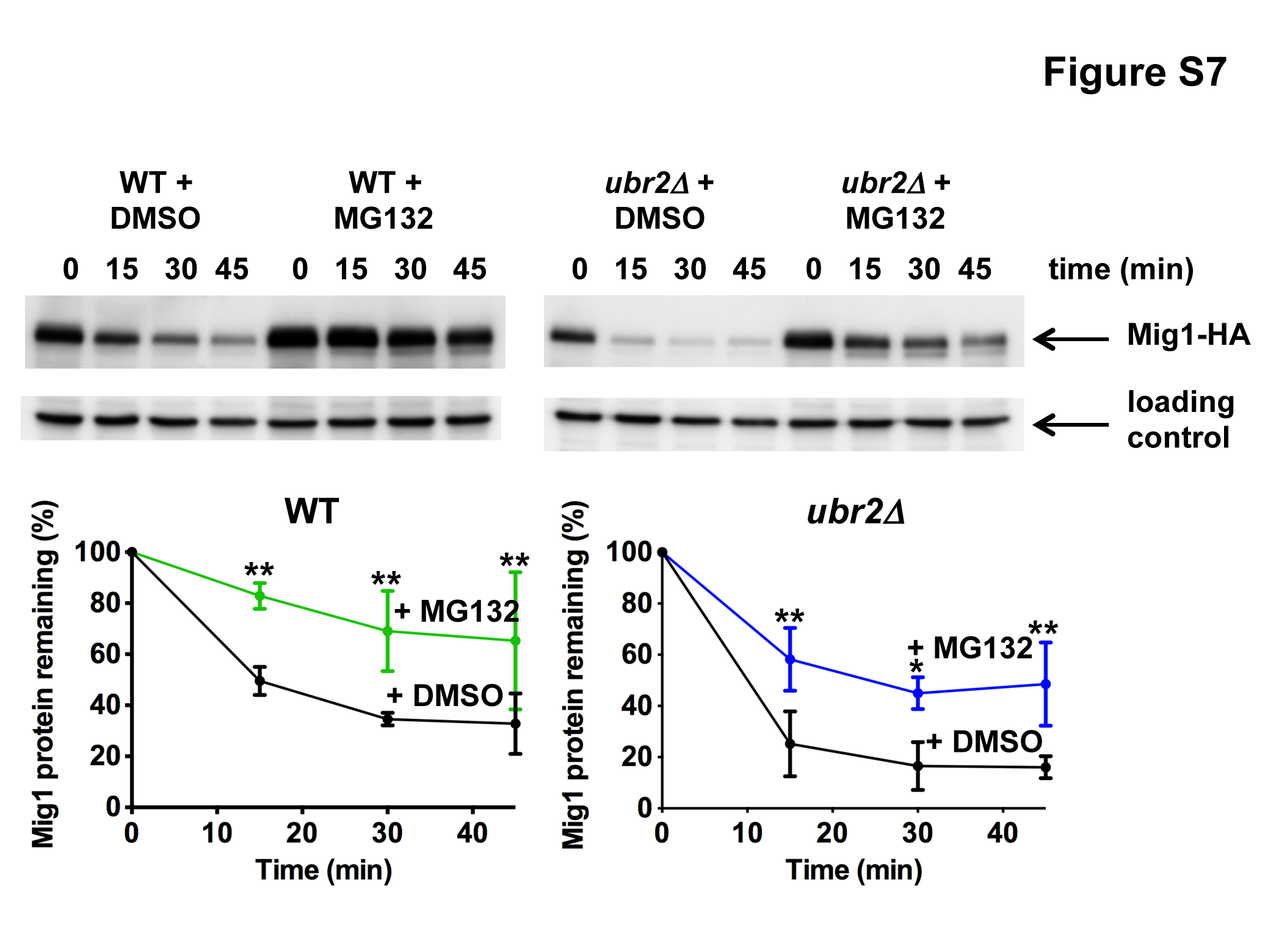

Supplement: S7 Fig — The turnover of Mig1 in cells grown in galactose was determined in WT and ubr2Δ cells after new synthesis was blocked with 200 μg/ml CHX in the absence or presence of the proteasome-specific inhibitor MG132. Mig1 levels were detected and quantified as described in Fig. 6. (TIF) [file pgen.1004968.s010.tif]

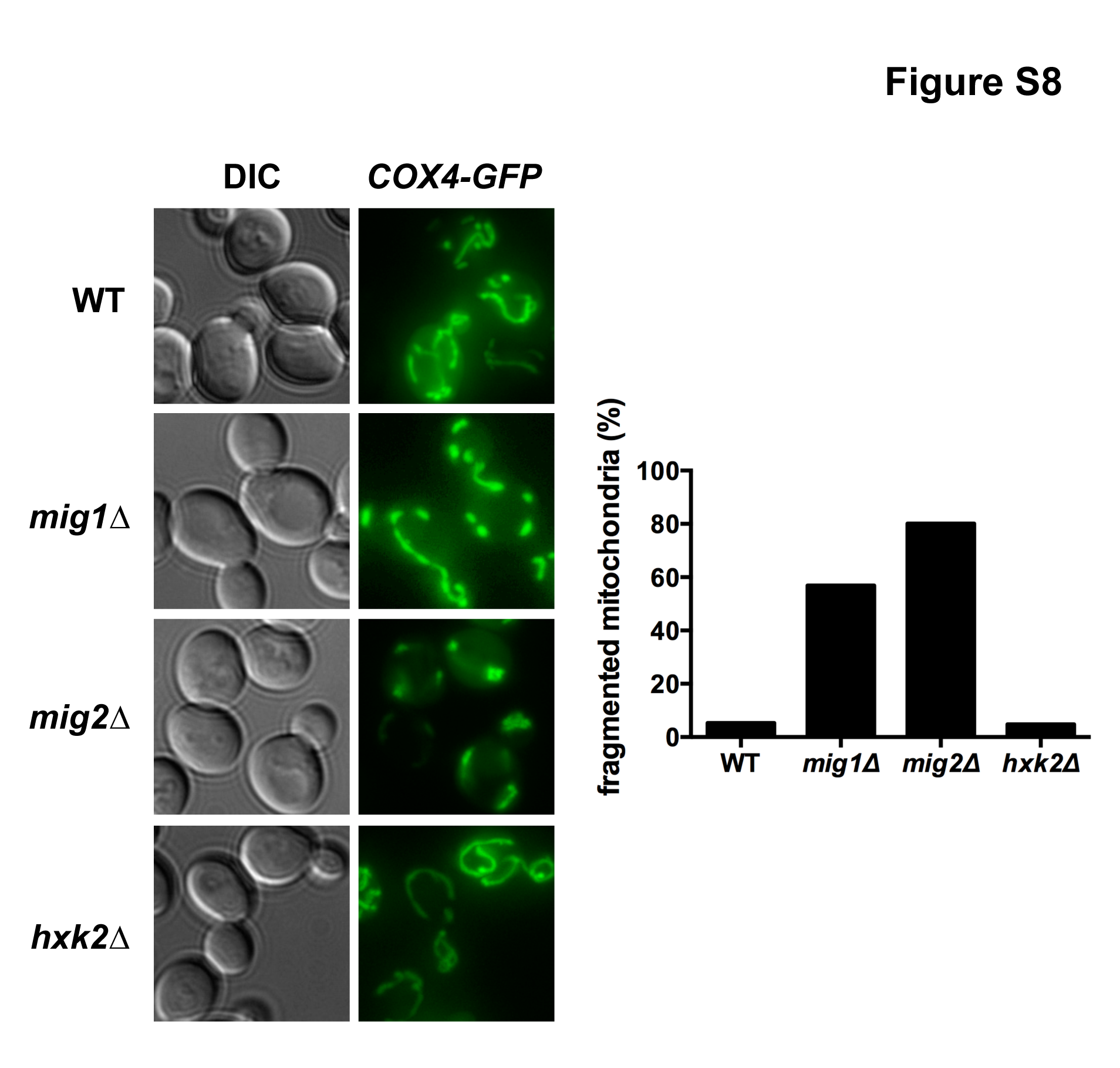

Supplement: S8 Fig — Loss of MIG1 induces mitochondrial hyperfragmentation. To visualize mitochondria, GFP was tagged with a mitochondrial import signal. The vector was introduced in cells deleted for MIG1, MIG2 or HXK2. Mitochondrial morphology was recorded via live cell fluorescence microscopy under logarithmic growth conditions. Projected sequential Z-stacks fluorescence images are presented. DIC: differential interference contrast. ~ 240 cells of each strain were analyzed by visual inspection (left panel). (TIF) [file pgen.1004968.s011.tif]

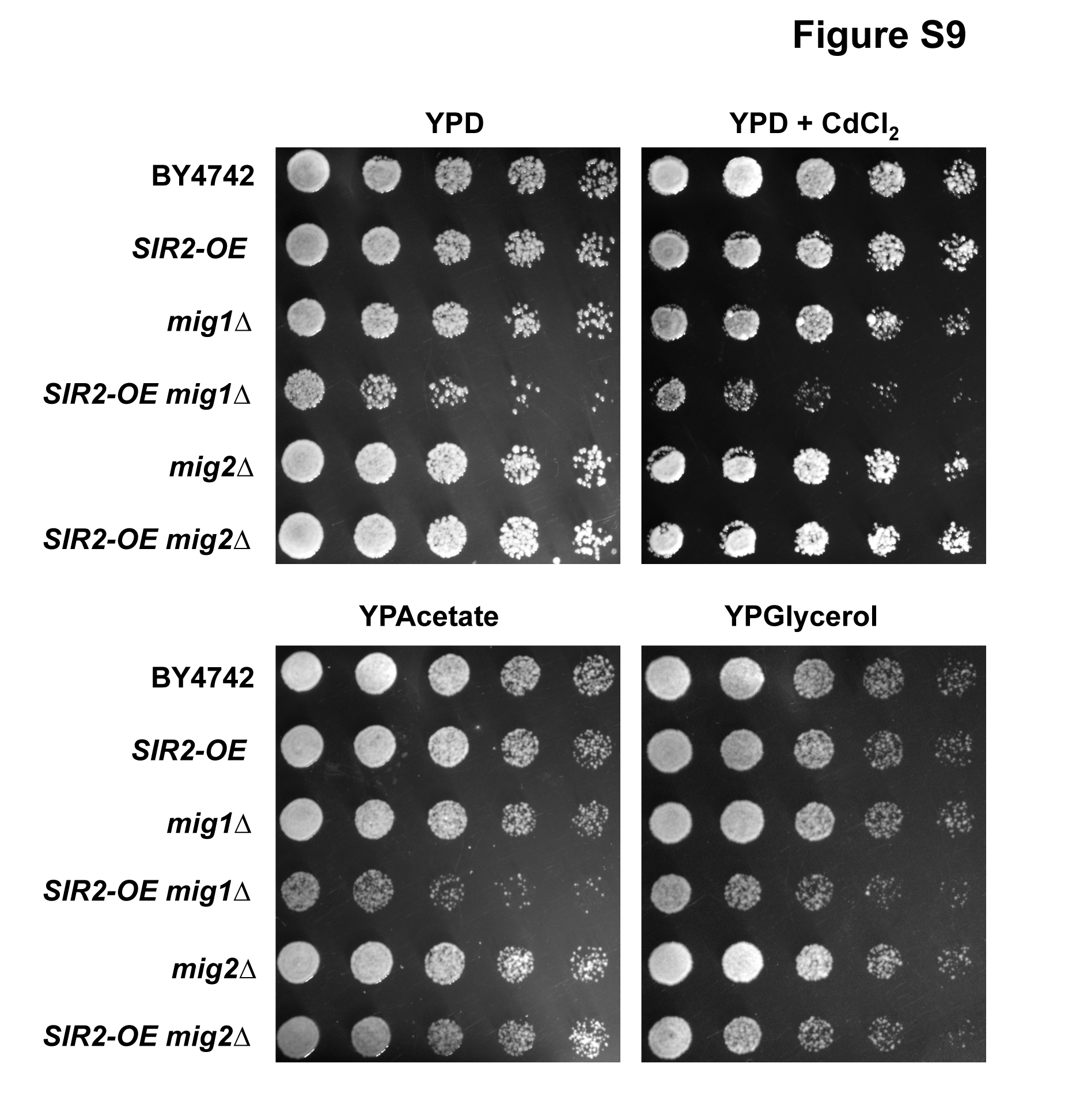

Supplement: S9 Fig — Phenotypic analysis of SIR2 overexpression strains in the absence of MIG1 or MIG2. 5-fold serial dilutions of the strains indicated were spotted on YPD, YPD supplemented with cadmium chloride (upper panels) or on complete media with acetate or glycerol as the sole carbon source (lower panels) and incubated at 30°C. (TIF) [file pgen.1004968.s012.tif]
